# Mechanical characterization of an incompressible, strain-hardening, transversely isotropic material

**DOI:** 10.1016/j.actbio.2025.10.062

**Published:** 2025-11-03

**Authors:** Qifeng Wang, Sheng Wang, Mahdi Ebrahimkhani, Thomas J. Royston, Eric J. Perreault, Kenneth R. Shull

**Affiliations:** a Department of Materials Science and Engineering, Northwestern University, 2220 Campus Drive, Evanston, 60208, IL, USA; b Department of Biomedical Engineering, Northwestern University, 2145 Sheridan Road, Evanston, 60208, IL, USA; c Shirley Ryan AbilityLab, 355 E Erie Street, Chicago, 60611, IL, USA; d Richard and Loan Hill Department of Biomedical Engineering, University of Illinois Chicago, 851 South Morgan Street, Chicago, 60607, IL, USA; e Department of Physical Medicine and Rehabilitation, Northwestern University, 710 N. Lake Shore Drive, Chicago, 60611, IL, USA

**Keywords:** Transversely isotropic, Strain energy, Finite element analysis, Indentation, Shear wave elastography

## Abstract

A strain energy approach for the characterization of strain-hardening, transversely isotropic materials was developed and validated through a combination of indentation and uniaxial extension experiments. These experiments were utilized because they can also be applied to directly measure the mechanical properties of many living tissues, including muscle. Model materials with transversely isotropic mechanical properties broadly representative of biological tissues were utilized in the experiments. These organogels were made from acrylic triblock copolymer solutions with an aligned cylindrical domain morphology. The strain energy function used here was proposed recently by Hegde *et al.*, and is based on the three independent linear elastic constants for a transversely isotropic material, along with two additional strain-hardening parameters. These five parameters were determined for the model material by indentation with a blade indenter aligned both parallel and perpendicular to the unique axis of the gel, and by uniaxial extension of the material along the directions parallel and perpendicular to the unique axis. The effect on the indentation curves of an applied tensile pre-stress applied along the unique axis was also investigated. Finite element modeling was used to generate interpolated functions that allow the elastic constants, along with their uncertainty, to be obtained from the experimental data in a straightforward manner. These parameters were then used to predict the wave speeds in pre-stressed material that would be measured by shear wave elastography, a commonly used technique for non-invasively characterizing the mechanical properties of biological tissues.

## 1. Introduction

Transversely isotropic materials have unique properties in the direction of a symmetry axis, with isotropic properties within the plane perpendicular to this axis. If the shear moduli are substantially less than the bulk compressive modulus, then the materials can be approximated as incompressible, with deformation occurring at constant volume. A range of biological tissues can be treated as incompressible transversely isotropic materials including skeletal muscle [1], tendon [2] and various structures in the central nervous system, such as the brainstem [3] and white matter in the cortex [4,5].

One important technique that has been used to characterize biological tissues is mechanical indentation, as summarized in the recent review article by He et al. [6] Indentation directly probes the stiffness of the materials and is complementary to methods based on wave propagation. The situation is quite complex for anisotropic materials, and a variety of indentation approaches have been used to quantify their relevant elastic constants. These techniques require that additional information be obtained apart from the single load/displacement curve that is typically obtained from an indentation experiment. In the work of Moghaddam et al. for example, the authors extract information about the mechanical anisotropy by measuring the aspect ratio of the elliptical contact that forms when a spherical indenter is brought into contact with an anisotropic material [7]. Liao and Xie obtained additional information by using a spherical indenter to probe the properties of a transversely isotropic material that had been cut at different angles with respect to the symmetry axis [8]. For complex samples like actual biological tissues it is generally easier to obtain the additional required information by using an asymmetric indenter and changing its orientation with respect to the sample. This is the approach taken by Bischoff et al. [9] Namani et al. [10] and Feng et al. [4,5] and is also the approach taken in our own work. Here we also account for the effect of a tensile stress applied along the symmetry axis on the measured indentation response. The effect of this stress is particularly important for muscle tissue, and has not been considered in previous indentation experiments. Because biological tissues are difficult to characterize directly, especially in situations where living tissues are being probed, it is very useful during the technique development stage to work with a synthetic ‘phantom’ that mimics the mechanical response of the tissues of interest. Sano et al. have provided a review of efforts to produce anisotropic synthetic hydrogels in the relevant property range [11]. In general terms, anisotropy is obtained by using a material with intrinsic anisotropy at the nanoscale or microscale, using an applied field of some sort to macroscopically align this underlying structure in a preferred direction. Examples of the intrinsic anisotropy include nanocrystalline domains in a polyvinyl alcohol hydrogel [12], cellulose nanocrystals [13], cellulose nanofibers [14], Spandex fibers [15], and polymers with liquid crystalline order [16]. In most cases the applied field resulting in the macroscopic alignment of these structures is a strain field, but other examples include a temperature gradient leading to directional solidification [13].

In this work we develop a synthetic model system based on a block copolymer gel with a cylindrical domain morphology, oriented by shear. The material is a self-assembling system that is quite easy to work with, and is highly elastic, with negligible viscoelastic character under quasi-static conditions [17,18]. It is a useful model elastic system for validating different experimental methodologies for characterizing a transversely isotropic material. These model materials strain harden at relatively low strains, and a strain energy function that accounts for this strain hardening is utilized. We utilize the strain energy function proposed recently by Hegde et al. which accounts for strain hardening in a realistic way and is written in terms of the independent elastic constants for a transversely isotropic material [19]. This strain energy function is used to describe the elastic response of our model material as probed both by indentation and uniaxial extension. This same strain energy function is used to predict the wave speeds as they would be determined by shear wave elastography, a technique that is commonly used to quantify the elastic constants of soft tissues because of its non-invasive nature [20-28]. We note that the elastic analysis does not account for time-dependent effects including viscoelasticity and poroelasticty. While these effects are often important in biological materials, our elastic approach applies to materials with a predominantly elastic character, including muscle tissue [22,29,30]. In addition, this elastic analysis serves as a starting point for quantifying more complex time-dependent behavior [31-33].

Our goal with this work is to provide a self-contained description of the approach that can be used to determine the elastic constants and axial stress for the transversely isotropic materials from a suite of the most appropriate available experiments. We begin with a discussion of the underlying mathematical formalism in the following section. We then introduce the model transversely isotropic gel, and describe results obtained from both uniaxial extension and indentation measurements. Results are obtained from the indentation of the material under different tensile loading conditions to illustrate the role of uniaxial stress on the indentation results. Finite element modeling was used to interpret the indentation results. These modeling results are presented in a parameterized form that enables future indentation results to be quantitatively analyzed without performing additional finite element calculations. An uncertainty analysis connected with this approach allows us to quantify uncertainties in material properties originating from experimental error in the measured contact stiffnesses, a capability that is particularly important when analyzing data from biological samples. We conclude with a discussion of the predicted wave speeds for our material that would be obtained from shear wave elastography.

## 2. Theory: Deformation of a transversely isotropic material

### 2.1. Notation

**Fig. 1.**
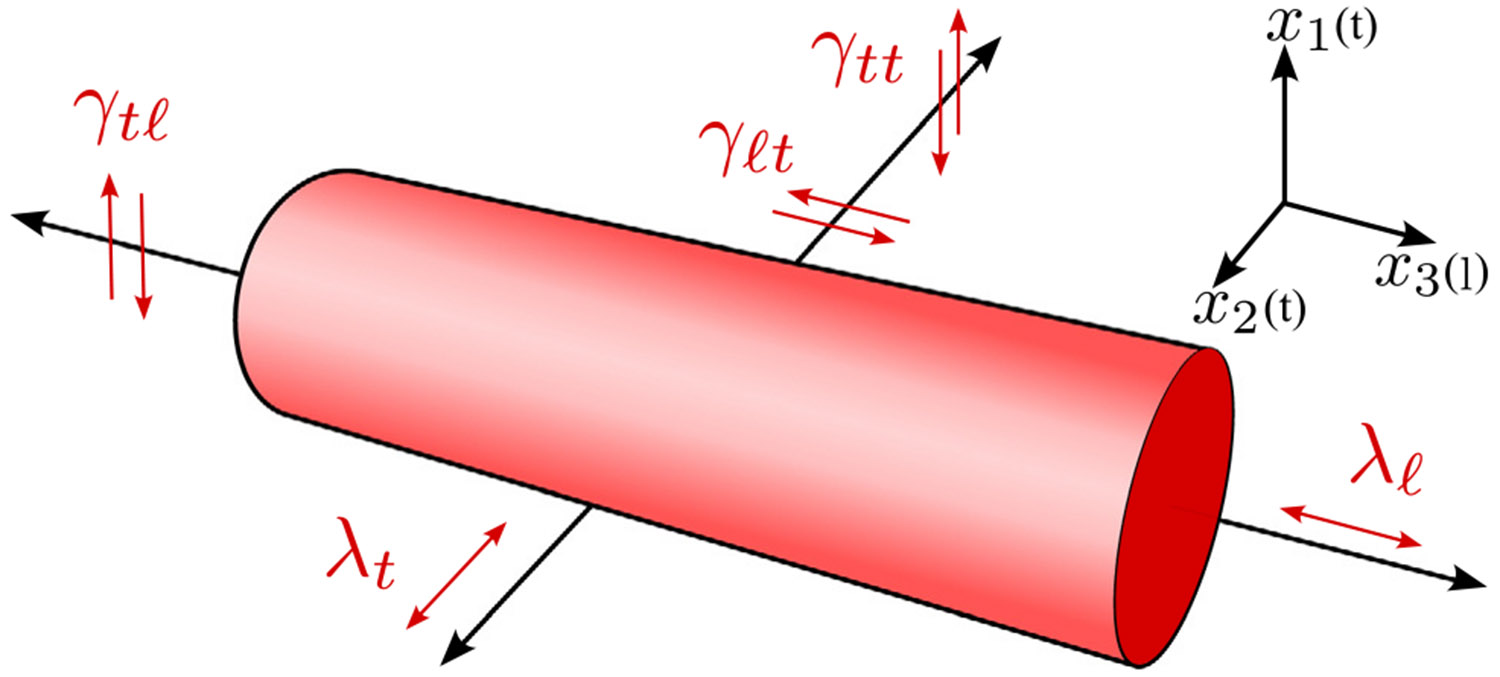
Transversely isotropic geometry illustrating different deformation modes.

Our notation for some of the relevant deformation modes for a transversely isotropic material is illustrated in Fig. 1. Transversely isotropic materials have a unique axis (the longitudinal axis, defined as the $x_{3}$ direction in our notation), with isotropic properties in the plane perpendicular to this (the $x_{1}-x_{2}$ plane in our notation). Several different deformation modes can be defined in terms of the following quantities used to characterize the different strains that are applied to the material:

- $\lambda_{\ell }$: Extension ratio for uniaxial extension of the material in the longitudinal direction.
- $\lambda_{t}$: Extension ratio for uniaxial extension of the material in the transverse direction.
- $\gamma_{tt}$: Shear strain for shear in the transverse plane.
- $\gamma_{t\ell }$: Shear strain for shear displacement in the transverse direction, with a displacement gradient in the longitudinal direction.
- $\gamma_{\ell t}$: Shear strain for shear displacement in the longitudinal direction, with a displacement gradient in the transverse direction.

Note that in the unstrained state the extension ratios describing the normal strains ($\lambda_{\ell }$ and $\lambda_{t}$) are equal to one, and the shear strains ($\gamma_{tt}$, $\gamma_{t\ell }$ and $\gamma_{\ell t}$) are all equal to zero.

Five independent elastic constants are required to specify the linear elastic behavior of materials with transversely isotropic symmetry, a number that is reduced to three if the material is incompressible. A variety of elastic constants can be chosen as the independent elastic constants, with one of them necessarily being the longitudinal shear modulus, $\mu_{L}$, for shear in a plane containing the longitudinal axis ($\gamma_{\ell t}$ or $\gamma_{t\ell }$ in Fig. 1) . The other two independent elastic constants are generally chosen from the following:

- $E_{L}$: The longitudinal Young’s modulus for extension in the longitudinal direction.
- $E_{T}$: The transverse Young’s modulus for extension in the transverse direction.
- $\mu_{T}$: Transverse shear modulus for shear in the transverse plane ($\gamma_{tt}$ in Fig. 1).
- $\nu_{tt}$: Poisson’s ratio in the transverse plane.

The possible pairs of the two other independent elastic constants besides $\mu_{L}$ and relationships between these different elastic constants for an incompressible, transversely isotropic material are listed in Table 1. Here we use $E_{L}$, $\mu_{T}$ and $\mu_{L}$ to specify the elastic properties of the material at small strains because these parameters appear directly in the strain energy function proposed by Hegde et al. [19], as described in more detail below.

### 2.2. Strain energy approach

The context of our work is in the refinement of methods to quantify the mechanical properties of muscle tissue. For this reason, we are not just interested in the linear elastic properties of the material, but also the non-linear properties, including the effects of strain hardening as the material is stretched in the longitudinal direction. An appropriate description of these materials requires a general approach based on the specification of the elastic strain energy of the material. The general approach is outlined in a variety of previous publications, including the thorough treatment of Ogden [34]. The details of our approach are quite similar to the more recent work of Rouze et al. in their description of shear wave propagation in hyperelastic materials that are isotropic in the unstrained state [35]. Here we extend this treatment to materials that are transversely isotropic in the absence of an applied strain.

#### 2.2.1. Specification of the strain state

Our starting point is the deformation gradient tensor, F, which describes the displacement gradients for a given strain state. We are interested in materials that are pre-stretched in the longitudinal direction (the $x_{3}$ direction in our case), quantified by the extension ratio, $\lambda_{\ell }$. For an incompressible material, where the principal extension ratios must multiply to 1, this deformation is described by the following deformation gradient tensor, $F_{0}$:

(1) $$F_{0}=\left[\begin{array}{ccc} 1∕\sqrt{\lambda_{\ell }} & 0 & 0\\ 0 & 1∕\sqrt{\lambda_{\ell }} & 0\\ 0 & 0 & \lambda_{\ell } \end{array}\right]$$

Subsequent to the application of this extension deformation, we apply small incremental shear strains to the material. Experimentally, this shear deformation could be associated with the excitation and propagation of shear waves through the material. Consider, for example, the case where the incremental deformation corresponds to some combination of the shear modes illustrated in Fig. 1, where the relevant shear strains are $\gamma_{tt}$, $\gamma_{t\ell }$ and $\gamma_{\ell t}$. In this case we have the following gradient tensor for this incremental deformation:

(2) $$F_{inc}=\left[\begin{array}{ccc} 1 & \gamma_{tt} & \gamma_{t\ell }\\ 0 & 1 & 0\\ 0 & \gamma_{\ell t} & 1 \end{array}\right]$$

The overall deformation gradient for an extensional deformation described by Eq. (1) followed by the shear deformations described by Eq. (2) is obtained from the product of $F_{inc}$ and $F_{0}$:

(3) $$F=F_{inc}F_{0}=\left[\begin{array}{ccc} 1∕\sqrt{\lambda_{\ell }} & \gamma_{tt}∕\sqrt{\lambda_{\ell }} & \gamma_{t\ell }\lambda_{\ell }\\ 0 & 1∕\sqrt{\lambda_{\ell }} & 0\\ 0 & \gamma_{\ell t}∕\sqrt{\lambda_{\ell }} & \lambda_{\ell } \end{array}\right]$$

In the specification of the strain energy functions, it is often convenient to introduce the right Cauchy–Green Tensor, C, and the left Cauchy–Green tensor, B, both of which are obtained directly from F:

(4) $$C=F^{T}F$$

(5) $$B={\mathrm{FF}}^{T}$$

Here $F^{T}$ is the transpose of F.

#### 2.2.2. Specification of the strain energy function

A variety of strain energy functions can be used, most of which are functions of various strain invariants, $I_{1}$, $I_{2}$, $I_{3}$, *etc*. The first invariant is given by the sum of the squares of the principal stress ratios, $\lambda_{1}$, $\lambda_{2}$, and $\lambda_{3}$:

(6) $$I_{1}=\lambda_{1}^{2}+\lambda_{2}^{2}+\lambda_{3}^{2}$$

The simplest theories of non-linear elasticity for isotropic materials, are based only on $I_{1}$. The most basic of these is the Neohookean model, for which the strain energy density for an incompressible material is given as follows :

(7) $$W_{NH}=\frac{\mu }{2}\;(I_{1}-3)$$

Here we use the ‘NH’ subscript to refer to this as the Neohookean model and also recognize that there is only one independent elastic constant, in this case expressed as the shear modulus, μ, for an incompressible isotropic material. More complicated strain energy functions are needed to describe the elastic properties of transversely isotropic materials with unique properties along the longitudinal axis. This is generally done by including some combination of the fourth and fifth strain invariants, $I_{4}$ and $I_{5}$ in the strain energy function [4,5]. These quantities are not formally invariants since they rely on the use of a coordinate system that is tied to the symmetry axis of the material, but they are invariant to rotations of the coordinate system around this axis. They are defined as follows:

(8) $$I_{4}=m\cdot (\mathrm{Cm})$$

(9) $$I_{5}=m\cdot (C^{2}m)$$

Here m is a unit vector directed along the longitudinal axis. In our coordinate system we fix. $x_{3}$ to the longitudinal direction, so we have:

(10) $$m=\left[\;\begin{array}{c} 0\\ 0\\ 1 \end{array}\;\right]$$

Most proposed strain energy functions for transversely isotropic materials include $I_{4}$ but not necessarily $I_{5}$. Feng et al. [4] and Murphy [36] have pointed out that in order to capture the linear elastic properties of the material with independent distinct values for $\mu_{L}$ and $\mu_{T}$ it is necessary to include both $I_{4}$ and $I_{5}$ in the strain energy function. Based on a combination of this work and the earlier work of Humphrey and Yin [37], Hegde et al. have developed a strain energy function for a transversely isotropic material that they refer to as the HYM model [19]. In the incompressible limit the HYM strain energy function can be written in terms of $E_{L}$, $\mu_{T}$ and $\mu_{L}$, in addition to two parameters, $c_{2}$ and $c_{4}$ that describe the strain hardening:

(11) $$W=\frac{\mu_{T}}{2c_{2}}\left[e^{c_{2}(I_{1}-3)}-1\right]+\frac{E_{L}+\mu_{T}-4\mu_{L}}{2c_{4}}\left[e^{c_{4}{\left(I_{4}^{1∕2}-1\right)}^{2}}-1\right]\;\;+\frac{\mu_{T}-\mu_{L}}{2}\;(2I_{4}-I_{5}-1)$$

Note that the Neohookean expression for W (Eq. (7)) is recovered for $c_{2}\;=\;c_{4}\;=\;0$ and $\mu_{T}=\mu_{L}=E_{L}∕3$. The first term has been shown to accurately describe the strain-hardening behavior of a series of isotropic synthetic and bio-based gels [38] and Hegde et al. used the full HYM model to describe the behavior of a range of incompressible, transversely isotropic materials [19]. The appeal of Eq. (11) is that it reduces to the appropriate linearized form characterized by three independent elastic constants necessary to describe a transversely isotropic material, while including parameters to describe isotropic strain hardening (through $c_{2}$) and the much stronger expected strain hardening for deformation along the symmetry axis (through $c_{4}$). While the approach we describe below is generalizable to other strain energy functions, we feel that the HYM function used here provides the best combination of accuracy and simplicity. The full Cauchy stress (true stress) tensor, σ, resulting from the deformation of a material with a strain energy function that depends only on $I_{1}$, $I_{4}$ and $I_{5}$ is obtained from the following expression [34]:

(12) $$\sigma =-pI+2\frac{\partial W}{\partial I_{1}}B+\frac{\partial W}{\partial I_{4}}\mathrm{Fm}⊗\mathrm{Fm}+2\frac{\partial W}{\partial I_{5}}\;(\mathrm{Fm}⊗\mathrm{BFm}+\mathrm{BFm}⊗\mathrm{Fm})$$

Here I is the identity matrix, ⊗ indicates the tensor product and p is a hydrostatic pressure term associated with the incompressibility constraint, obtained from the appropriate boundary conditions. In some cases the relevant quantities are incremental moduli describing the stiffness of the material that has already been deformed, in our case by uniaxial tension applied along the longitudinal axis. The relevant incremental shear moduli are obtained by differentiating the appropriate shear stress with respect to the corresponding applied incremental strain. In this way we define three distinct incremental shear moduli, $\mu_{tt}$, $\mu_{t\ell }$ and $\mu_{\ell t}$, all of which are functions of the pre-stretch, $\lambda_{\ell }$. In our notation, where the $x_{3}$ direction is the longitudinal direction and the $x_{1}-x_{2}$ plane is the transverse plane, we have:

(13) $$\mu_{tt}(\lambda_{\ell })=\frac{\partial \sigma_{12}(\lambda_{\ell },\gamma_{tt})}{\partial \gamma_{tt}};\mu_{t\ell }(\lambda_{\ell })=\frac{\partial \sigma_{23}(\lambda_{\ell },\gamma_{t\ell })}{\partial \gamma_{t\ell }};\mu_{\ell t}(\lambda_{\ell })=\frac{\partial \sigma_{23}(\lambda_{\ell },\gamma_{\ell t})}{\partial \gamma_{\ell t}}$$

In the calculations for a given shear mode ($\gamma_{tt}$ for example) the other shear strains ($\gamma_{t\ell }$ and $\gamma_{\ell t}$ in this example) are set to zero in Eq. (3). Note that in the unstrained limit, the incremental shear moduli are given by the appropriate shear modulus, either $\mu_{T}$ or $\mu_{L}$:

(14) $$\;\mu_{tt}\;(\lambda_{\ell }\to 1)=\mu_{T}\;\mu_{\ell t}\;(\lambda_{\ell }\to 1)=\mu_{t\ell }\;(\lambda_{\ell }\to 1)=\mu_{L}$$

As the material is deformed in the longitudinal direction and a tensile stress, $\sigma_{\ell }$, develops in the longitudinal direction, the tℓ and ℓt shear modes are no longer equivalent, so that in general $\mu_{t\ell }\neq \mu_{\ell t}$. This result is very useful, providing a method for determining the tensile stress within the material, according to the following equation, derived by Biot [39] and explained more generally by Destrade and Ogden [40] (Eq. 6.21 in Destrade and Ogden):

(15) $$\mu_{t\ell }-\mu_{\ell t}=\sigma_{\ell },$$

Note that while the stress dependence of the various incremental moduli depend on the details of the strain energy function, Eq. (15) holds more generally.

We also obtain incremental extensional moduli, defined as $E_{\ell \ell }$ in the longitudinal direction and $E_{tt}$ in the transverse direction. Calculation of $E_{\ell \ell }$ is determined in straightforward way as the derivative of the normal stress in the longitudinal direction with respect to the corresponding extension, $\lambda_{\ell }$, with all shear components in Eq. (3) set to zero:

(16) $$E_{\ell \ell }(\lambda_{\ell })=\frac{\partial \sigma_{\ell }\;(\lambda_{\ell })}{\partial \lambda_{\ell }}$$

Young’s modulus for extension along the longitudinal axis, $E_{L}$ is simply given by the limiting value of $E_{\ell \ell }$ for $\lambda_{\ell }=1$. A similar expression applies for the determination of $E_{tt}$, the differential modulus for uniaxial extension of the material in the transverse direction. Defining the 1 axis as the axis of deformation, with $\sigma_{t}\equiv \sigma_{11}$, we have:

(17) $$E_{tt}(\lambda_{t})=\frac{\partial \sigma_{t}\;(\lambda_{t})}{\partial \lambda_{t}}$$

The case of transverse extension is more complicated than the case of longitudinal extension because the material is no longer isotropic in the plane perpendicular to the applied extension. By defining β as the ratio of extension in the 3 and 2 directions, and remembering that the product of the three principal extension must be unity for an incompressible material, we obtain the following for the deformation gradient tensor for transverse extension:

(18) $$F=\left[\begin{array}{ccc} \lambda_{t} & 0 & 0\\ 0 & \sqrt{\frac{1}{\beta \lambda_{t}}} & 0\\ 0 & 0 & \sqrt{\frac{\beta }{\lambda_{t}}} \end{array}\right]$$

For low strains β is determined by the elastic constants of the material:

(19) $$\beta =1+(\lambda_{t}-1)\left(1-\frac{4\mu_{T}}{E_{L}+\mu_{T}}\right)$$

For higher strains the value of β may deviate slightly from the value given by Eq. (19), and is given by the requirement that the lateral stresses vanish. The equations written in this section can be used to obtain expressions for the stress components and the different moduli in terms of the applied deformation, $\lambda_{\ell }$, and the 5 independent parameters appearing in the strain energy function ($\mu_{T}$, $\mu_{L}$, $E_{L}$, $c_{2}$ $c_{4}$). These expressions are rather cumbersome when written out fully, so we do not do this here. Fortunately, the equations can be readily manipulated with the symbolic math capabilities of modern programming tools, in our case Python. Scripts used to generate all of the figures in this paper are included with the supporting information, as are some of the more important expressions generated by these scripts.

A variety of elastic constants have been defined in this section, and it is useful to keep the following features in mind:

- Elastic constants with a single subscript ($\mu_{T}$, $\mu_{L}$, $E_{L}$) describe the linearly elastic behavior of the undeformed material.
- Elastic constants with two subscripts ($\mu_{tt}$, $\mu_{\ell t}$, $\mu_{t\ell }$, $E_{tt}$, $E_{\ell \ell }$) are differential moduli that describe the slope of the stress/strain curve for a specified strain state and deformation mode.

## 3. Materials and methods

### 3.1. Model material: Transversely isotropic organogel

**Fig. 2.**
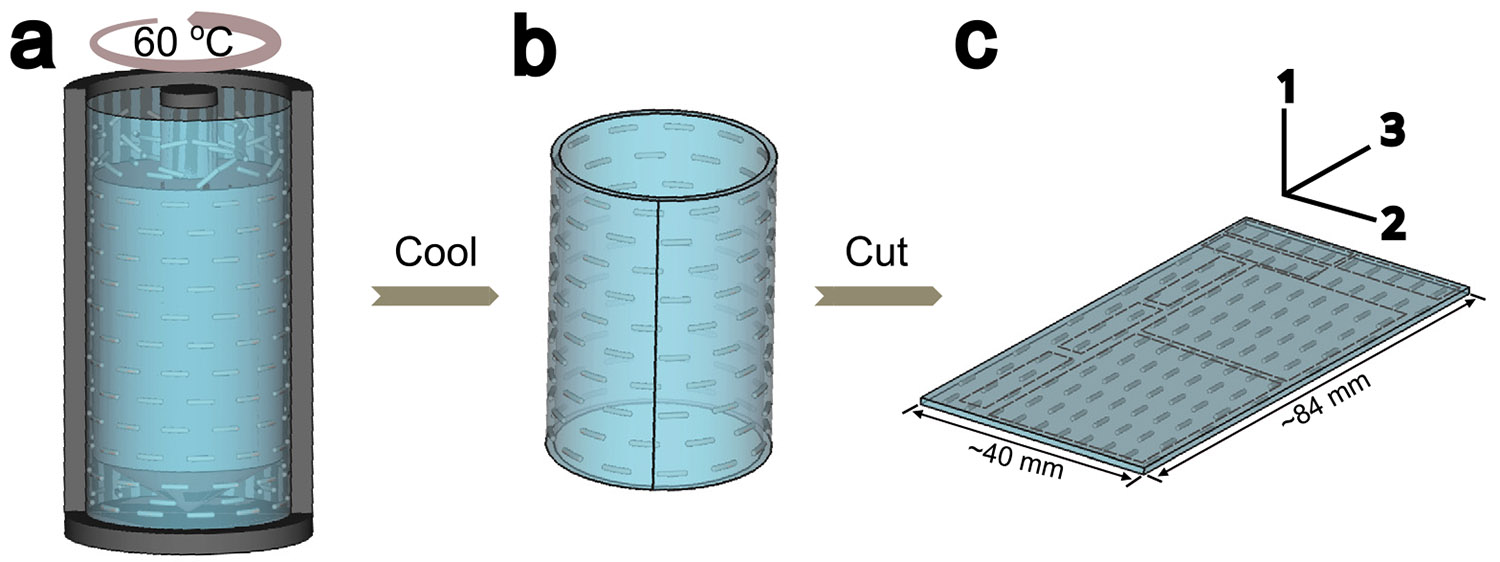
Procedure for the formation of a model, transversely isotropic gel. (a) Alignment of the micelle solution of cylindrical micelles in a liquid mixture at 60 °C by constant shear; (b) Quenching to room temperature to freeze in the aligned structure; (c) Removal of a flat sheet with a thickness of 1.1 mm from the central portion of the sample. The dashed lines in (c) illustrate the ideal cuts for the samples used in the presented work. Tensile tests were performed on the smaller rectangular cuts with a width of 5 mm and the indentation experiments were performed in the central regions of the larger rectangular cuts.

A poly(methyl methacrylate)-poly(n-butyl acrylate)- poly(methyl methacrylate) (PMMA-PnBA-PMMA) triblock copolymer was provided by Kuraray, Japan. The polymer has a total molecular weight of 77 kDa with an overall PMMA weight fraction of 0.60. The gel was formed as a 2-ethyl-hexanol (Sigma-Aldrich, ≥ 99.6%) solution with an overall PMMA end-block concentration of 22 wt%, initially heated to ≈ 90 °C to produce a polymer solution that is homogeneous at the molecular scale. As the temperature is cooled the PMMA end-blocks aggregate to form an elastic gel, characterized by a relaxation time that increases dramatically as the temperature is cooled and the PMMA aggregates become glassy [17]. The specific concentration was chosen to produce a cylindrical micelle geometry [18] that can be aligned by shear. The general procedure for making the anisotropic samples is illustrated in Fig. 2. A hot solution of the triblock copolymer was poured into a couette cell and slowly cooled while a continuous shear flow was applied to the sample using a rheometer. The shear flow was stopped when gelation of the sample caused the torque applied to the sample to approach the torque limit of the rheometer. The gel was then removed from the rheometer and cut up into smaller sections as needed. The longitudinal direction (the $x_{3}$ direction) corresponds to the direction of the applied shear. In this way we obtain a strain-hardening transversely isotropic material that mimics many of the mechanical features of muscle tissue.

### 3.2. Indentation

**Fig. 3.**
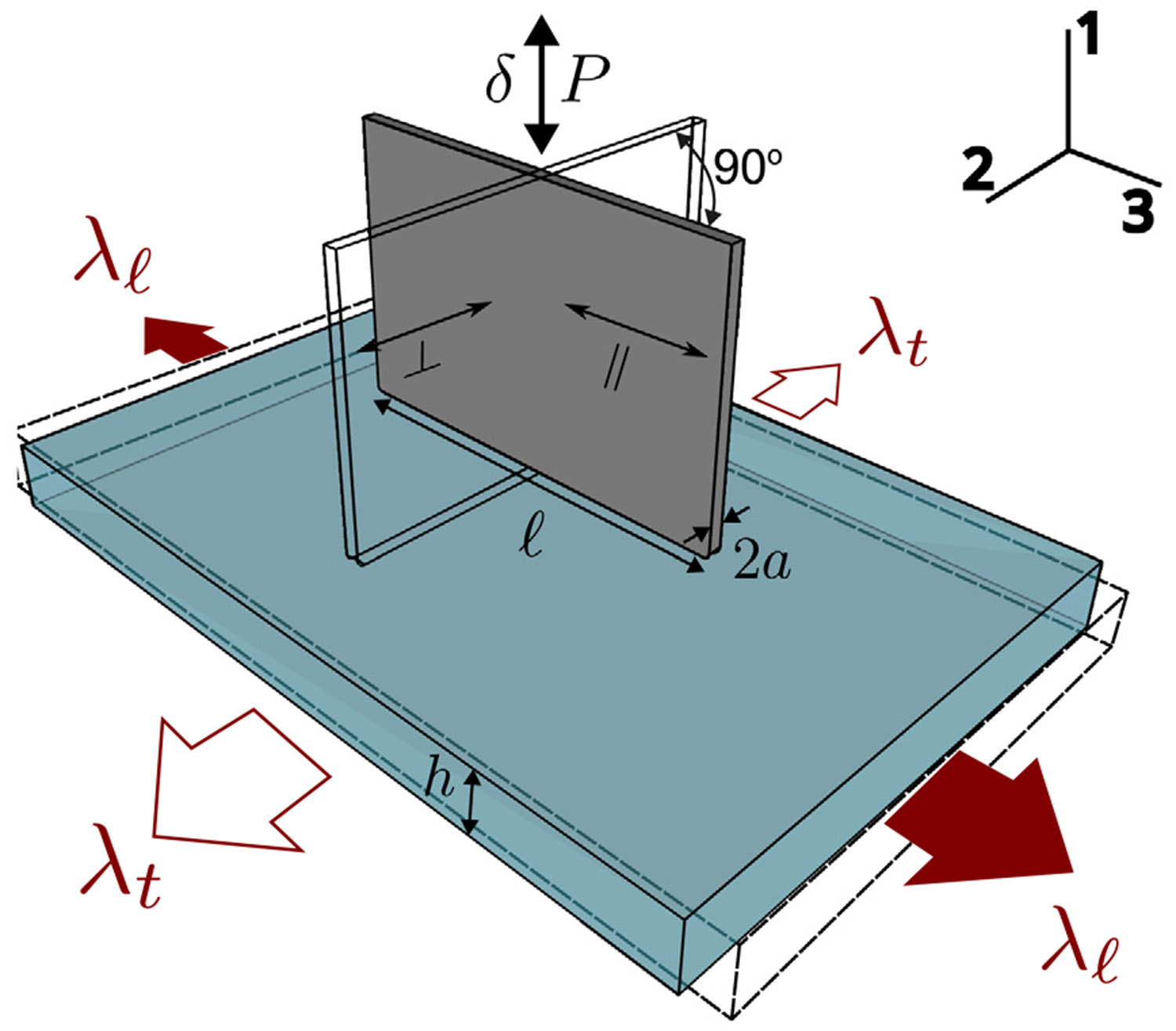
Schematic illustration of the indentation geometry. In our case the probe length, ℓ is 20 mm, the probe half-width, a, is 0.07 mm, and the gel thickness, h, is 1.1 mm.

The indentation geometry is illustrated in Fig. 3. The rectangular contact surface of the indenter is flat with a length, ℓ, of 20 mm, and a half-width, a, of 0.07 mm. The anisotropy of the elastic properties was quantified by aligning the long axis of the indenter parallel or perpendicular to the symmetry axis of the gel and measuring the applied load, P, as a function of displacement, δ, in each case. The effect of pre-strain was investigated applying an axial extension ratio $\lambda_{\ell }$ to the gel prior to the indentation. The nominal stress, $\sigma_{N}$ is obtained by dividing the load by the indenter cross section:

(20) $$\sigma_{N}=\frac{P}{2\ell a}$$

The ratio of the displacement, δ to the half-width, a, gives an estimate of the strain. We define a contact modulus s, in the following way:

(21) $$s\equiv \frac{\partial \sigma_{N}}{\partial (\delta ∕a)}$$

The contact modulus defined in this way is more convenient to conceptualize and relate to the actual moduli than is the commonly used contact stiffness, S, given by ∂P∕∂δ. With the long edge of indenter parallel (∥) and perpendicular (⊥) to the longitudinal axis, we obtain values for two respective contact moduli, $s_{∥}$ and $s_{⊥}$. These contact moduli are used along with the tensile data to obtain the elastic constants as described below.

## 4. Results and discussion

Values of the different elastic constants determined from our experiments are listed in Table 2. The longitudinal Young’s modulus, $E_{L}$, is most sensitively determined by longitudinal extension of the gel. Similarly, the transverse shear modulus, $\mu_{T}$, is coupled to the parallel contact modulus, $s_{∥}$, in a straightforward way. The longitudinal shear modulus, $\mu_{L}$, has a more complicated relationship to the measured quantities, but can be extracted from the anisotropy in the contact modulus, once $E_{L}$ and $\mu_{T}$ are known. Our primary purpose in the following is to describe how the elastic constants are determined, and to parameterize our analysis so that finite element simulations do not need to be performed in the future in order to extract the elastic combinations from measurements of longitudinal extension, along with parallel and perpendicular indentation.

### 4.1. Tensile testing

**Fig. 4.**
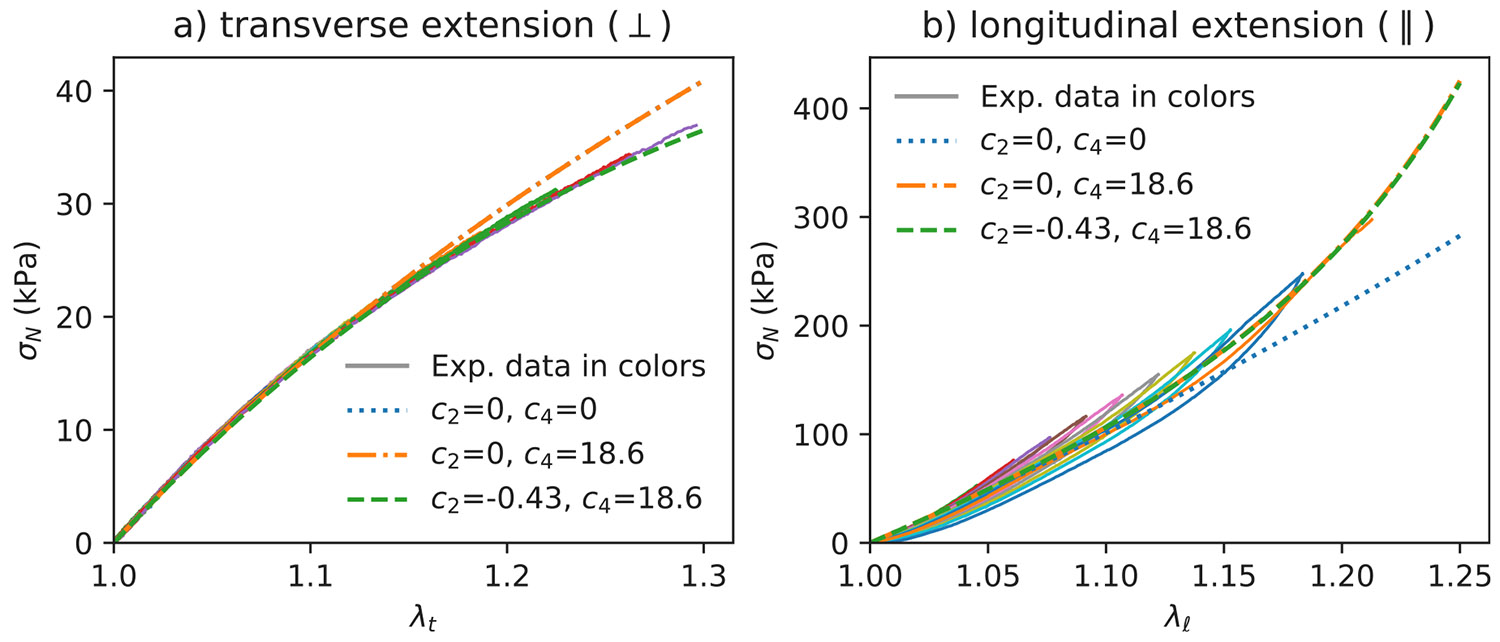
Nominal stress vs. extension ratio for the gels deformed in transverse extension (a) and longitudinal extension (b). The continuous extension tests on a single sample for each direction were measured with an increment of 0.2 mm maximum displacement each cycle. The dotted lines correspond to the predictions from Eq. (12), using the indicated values of $c_{2}$ and $c_{4}$ and the other material coefficients listed in Table 2. In part (a) the two predicted curves with $c_{2}=0$ overlap and in part (b) the two predicted curves with $c_{4}=18.6$ overlap.

Plots of the nominal stress (force divided by undeformed cross-sectional area) for one sample deformed in the transverse direction and one sample deformed in the longitudinal direction are shown in Fig. 4. For each sample we plot loading and unloading curves for increasing values of the maximum extension ratio. No hysteresis is observed for the transverse extension curves, whereas some mechanical hysteresis is observed for the longitudinal extension curves. We also plot the predictions of Eq. (12), using the parameters listed in Table 2. We note here that for an incompressible material deformed in uniaxial extension to an extension ratio, λ, the nominal tensile stress in the direction of extension is obtained by dividing the corresponding true stress by λ. Note that the model without strain hardening ($c_{2}=c_{4}=0$) fits the extension data well for strains up to about 15% ($\lambda_{\ell }=1.15$), but a nonzero value for $c_{4}$ is needed to predict the longitudinal extension data at higher strains. This difference in $c_{4}$ has no effect on the calculated stresses in transverse extension. Similarly, small changes in $c_{2}$ affect the stress prediction in transverse extension but not in longitudinal extension. Quantitative agreement with the full load curves in both transverse and longitudinal extension is obtained for the full range of measured extensions with $c_{2}=-0.43$ and $c_{4}=18.6$. Agreement is also obtained with $c_{2}=0$, indicating that addition of a single parameter ($c_{4}$) to the 3 linear elastic coefficients ($\mu_{T}$, $\mu_{L}$ and $E_{L}$) is generally sufficient to characterize the model gel over the range of strains that were investigated.

It is useful here to discuss the uncertainties in the values of $E_{L}$ and $\mu_{T}$ that are obtained from the indentation experiments. The uncertainty in $E_{L}$ is most straightforward, since the relative uncertainty in this quantity is the same as the relative uncertainty in the value of the slope of the longitudinal stress/strain curve at low strains. For our experiments we assume a 5% error in this slope, which gives the 5% error for $E_{L}$ listed in Table 2. This uncertainty in $E_{L}$ propagates to the determination of the uncertainty in $\mu_{T}$, which is related to both $E_{L}$ and $E_{T}$ in the following way (see Table 1):

(22) $$\mu_{T}=\frac{E_{T}E_{L}}{4E_{L}-E_{T}}$$

A 5% error in both $E_{T}$ and $E_{L}$ gives a 5.2% uncertainty in $\mu_{T}$. A linearized analysis was used to reach this conclusion, where the uncertainty of a function, y, of two variables, $x_{1}$ and $x_{2}$ is assumed to be related to the uncertainties in $x_{1}$ and $x_{2}$ as follows:

(23) $${\left(\delta y\right)}^{2}\approx {\left(\frac{\partial y}{\partial x_{1}}\delta x_{1}\right)}^{2}+{\left(\frac{\partial y}{\partial x_{2}}\delta x_{2}\right)}^{2}$$

In this particular example y is $\mu_{T}$, $x_{1}$ is $E_{L}$ and $x_{2}$ is $E_{T}$. Because $E_{L}\gg E_{T}$, $\mu_{T}$ is very nearly independent of $E_{L}$, with $\mu_{T}\approx E_{T}∕4$. For this reason the uncertainty in $E_{L}$ contributes very little to the uncertainty in $\mu_{T}$, and the measurement of the transverse modulus in extension is nearly equivalent to a more direct measurement of the transverse shear modulus.

While it is straightforward to measure the stress/strain curve for our model gels in transverse extension, this is not a geometry that can easily be applied in more complicated experimental scenarios. For example, in our own research these models are applied to muscle tissues that are being extended along the longitudinal axis. It is not feasible in this case to grip the sample in a way that enables a transverse extension experiment to be performed. Instead, we rely on indentation to determine the elastic constants as described in the following section. The transverse extension data for our model gels is included here as a consistency check on our use of the strain energy function and the elastic constants that are extracted from the indentation measurements.

### 4.2. Indentation

**Fig. 5.**
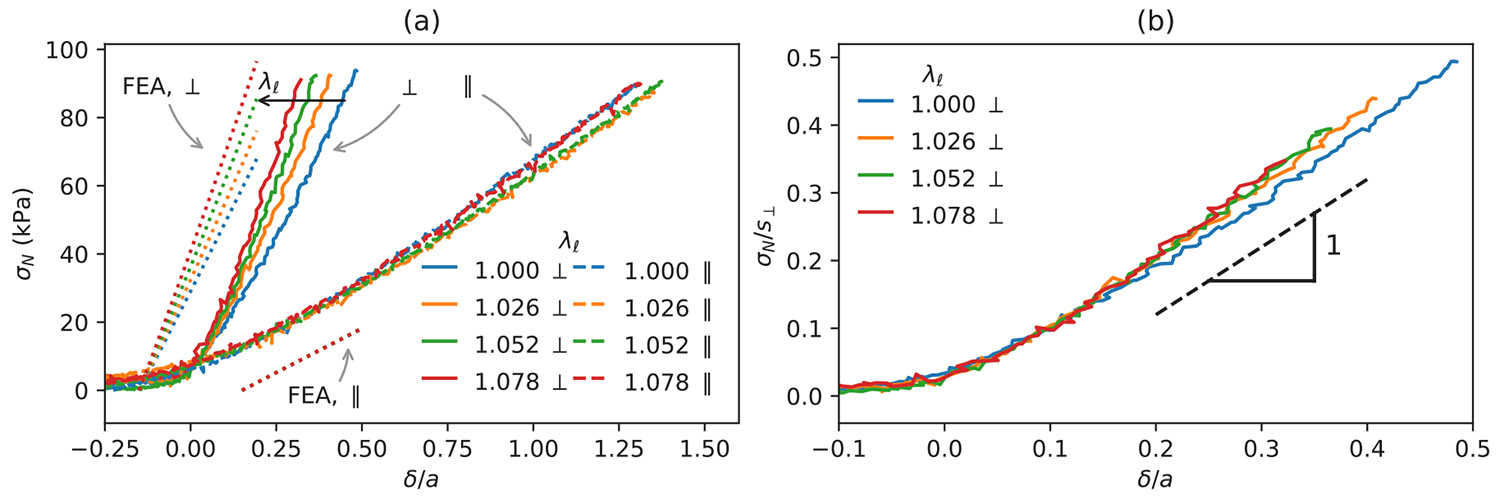
(a) Indentation curves for materials extended along the longitudinal axis and then indented with the long axis of the indenter aligned parallel (∥) or perpendicular (⊥) to the longitudinal direction. The dotted lines show the FEA simulated data (shifted ±0.15 horizontally for clarity) determined from the parameters in Table 2. (b) Data from the perpendicular indentation from part a, normalized by the values of $s_{⊥}$ from FEA simulation (values from ‘FEA with HYM’ column in Table 3). The dashed line in this case has a slope of 1, consistent with our definition of the contact modulus.

Indentation curves for the long axis of the indenter aligned parallel and perpendicular to the longitudinal axis are shown in Fig. 5a. A series of indentations were performed with longitudinal pre-strains, quantified by extension ratios ($\lambda_{\ell }$) ranging from 1 to 1.09. Our focus initially is on the sample with no pre-strain ($\lambda_{\ell }=1$). The most straightforward quantity to extract from the indentation data is the transverse shear modulus, $\mu_{T}$, which is related to $s_{∥}$ and the confinement ratio, a∕h, through the following equation:

(24) $$\frac{\mu_{T}}{s_{∥}}\approx -0.27\;\text{ln}\left(\frac{a}{2h}\right)$$

This expression is adapted from Ref. [41], with a simplified form to give a better fit to our own finite element data and reported results from other groups for hydrogels [10] and biological tissues [4,5]. Values of $\mu_{T}∕s_{∥}$ are plotted as a function of a∕h in the supporting information. These data include values of $E_{L}∕(3\mu_{T})$ ranging from 1 to 100 and values of $\mu_{L}∕\mu_{T}$ ranging from 1 to 100, but collapse nicely onto a single curve given by Eq. (24). This result originates from the fact that for a highly symmetric (ℓ≫a) indenter aligned parallel to the symmetry axis of the material, the deformation is dominated by transverse shear, allowing us to directly obtain from $\mu_{T}$ from $s_{∥}$. The uncertainty in a∕h does not contribute substantially to the uncertainty in $\mu_{T}$. The 10% uncertainty in the value of $\mu_{T}$ shown in Table 2 originates from our estimated 10% uncertainty in the measured value of $s_{∥}$.

**Fig. 6.**
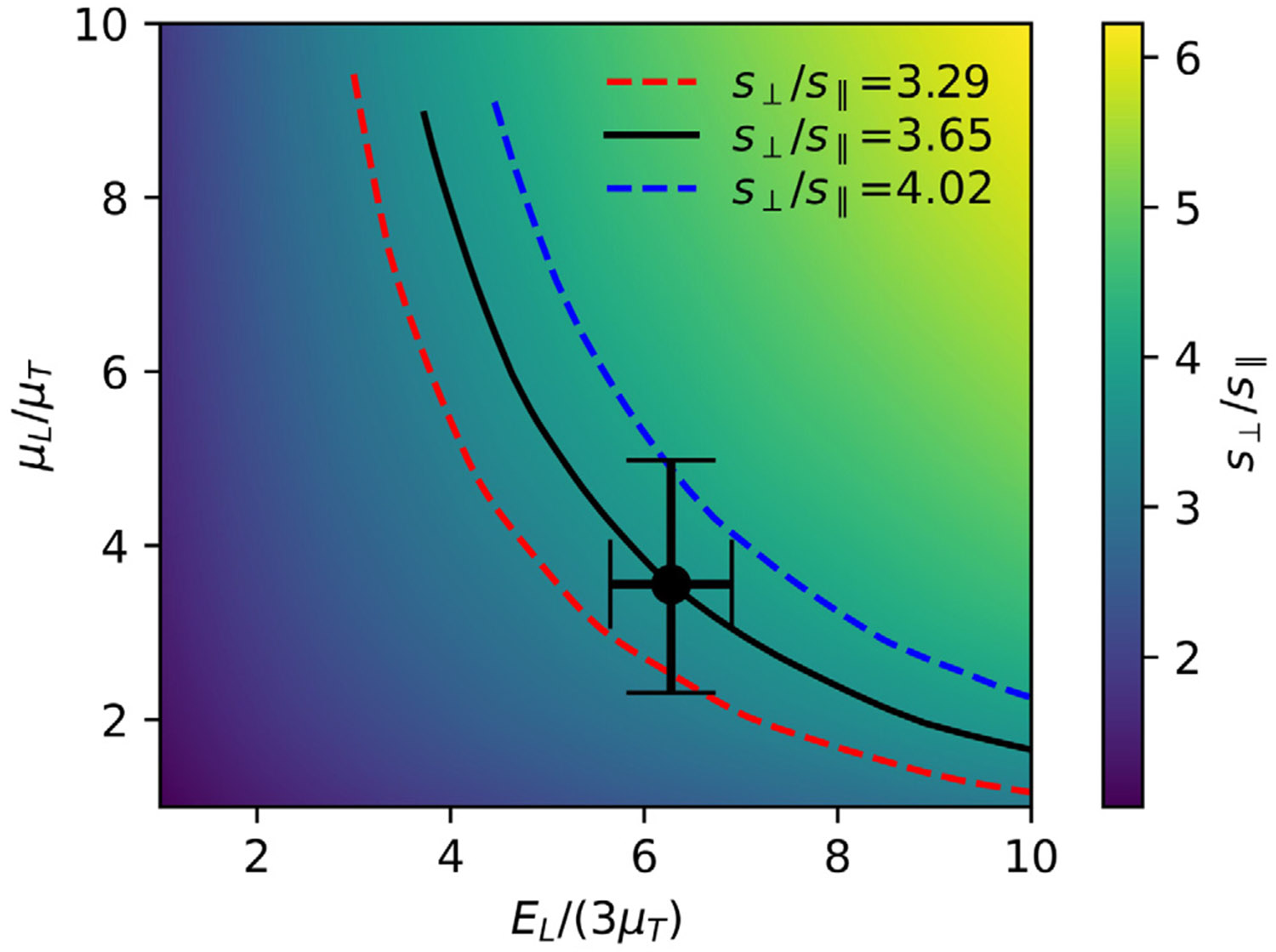
Color map illustrating the dependence of the contact stiffness ratio, $s_{⊥}∕s_{∥}$, on the two asymmetry parameters, $E_{L}∕(3\mu_{T})$ and $\mu_{L}∕\mu_{T}$. The lines are contours of constant $s_{⊥}∕s_{∥}$, corresponding to the values listed on the legend. The point corresponds a material with the properties listed in Table 2, with the error bar in $\mu_{L}∕\mu_{T}$ originating from an assumed 10% error in both $E_{L}∕(3\mu_{T})$ and $s_{⊥}∕s_{∥}$.

The value of $s_{⊥}$, the contact stiffness obtained when the indenter is oriented perpendicular to the symmetry axis, is more complicated and depends on all three independent moduli. Here we focus on the contact anisotropy ratio, $s_{⊥}∕s_{∥}$. Use of this quantity allows us to reduce the number of independent parameters from 3 to 2, since increasing all 3 elastic constants by the same multiplicative factor will not change the ratio of $s_{⊥}$ to $s_{∥}$. Here we perform a variety of simulations from which we obtain a smooth interpolated function for $s_{⊥}∕s_{∥}$ as a function Of $E_{L}∕(3\mu_{T})$ and $\mu_{L}∕\mu_{T}$. Note that both of these independent variables are equal to one for the isotropic case, where $s_{⊥}∕s_{∥}$ is also equal to one. An example for the geometric parameters relevant to our indentation experiments (a∕h=0.064 and ℓ∕a=400) is shown in Fig. 6. Here the x and y axes of the plot correspond to the two asymmetry parameters, $E_{L}∕(3\mu_{T})$ and $\mu_{L}∕\mu_{T}$, and the color map corresponds to the resultant value of the stiffness ratio, $s_{⊥}∕s_{∥}$.

The lines in Fig. 6 are contours of fixed $s_{⊥}∕s_{∥}$. Interpolated functions representing these curves (obtained from finite element data and Python scripts included in the supporting information) can be used to obtain $\mu_{L}$, once $E_{L}$ and $s_{⊥}$ are known, using a numerically determined function, $f_{1}$ that relates $\mu_{L}∕\mu_{T}$ to $E_{L}∕(3\mu_{T})$ and $s_{⊥}∕s_{∥}$:

(25) $$\frac{\mu_{L}}{\mu_{T}}=f_{1}\left(\frac{s_{⊥}}{s_{∥}},\frac{E_{L}}{3\mu_{T}}\right)$$

This function is determined from an interpolation of finite element data obtained over a wide range of values for both $s_{⊥}∕s_{∥}$ and $E_{L}∕(3\mu_{T})$. The relevant finite element data and the Python script used to generate $f_{1}$ are included in the supporting information.

The function $f_{1}$ is determined by interpolation of a set of finite element data obtained for the appropriate values of a∕h and ℓ∕a. This function enables us to determine the uncertainties in $\mu_{L}$, given the uncertainties obtained from the other parameters obtained from steps 1 and 2 above. We use Eq. (23) above to estimate the uncertainty, with $y=\mu_{L}∕\mu_{T}$, $x_{1}=s_{∥}∕s_{⊥}$ and $x_{2}=E_{L}∕(3\mu_{T})$. The error bars in Fig. 6 correspond to a 10% uncertainty in both $E_{L}∕(3\mu_{T})$ and $s_{∥}∕s_{⊥}$. While this approach is admittedly a simplification to what is a more complex multi-variable analysis, this simple estimation of the input uncertainties is consistent with the uncertainties in the individual parameters listed in Table 2. The resultant 40% uncertainty in $\mu_{L}∕\mu_{T}$, and hence in $\mu_{L}$ itself, is substantially larger than the uncertainties in the other parameters, illustrating the difficulty in accurately determining $\mu_{L}$ from an indentation experiment.

To summarize, the overall procedure for obtaining the 3 independent elastic constants characterizing the material response in the low-strain regime is as follows:

1. Obtain $E_{L}$ from a tensile measurement along the longitudinal axis.
2. Obtain the contact stiffnesses $s_{∥}$ and $s_{⊥}$ from indentation measurements with the long axis of the indenter parallel and perpendicular to the symmetry axis.
3. Use Eq. (24) to obtain $\mu_{T}$ from $s_{∥}$.
4. Use the function $f_{1}$ to obtain $\mu_{L}$ from $\mu_{T}$, $E_{L}$, $s_{⊥}$ and $s_{∥}$.

### 4.3. Effect of pre-stretch

**Fig. 7.**
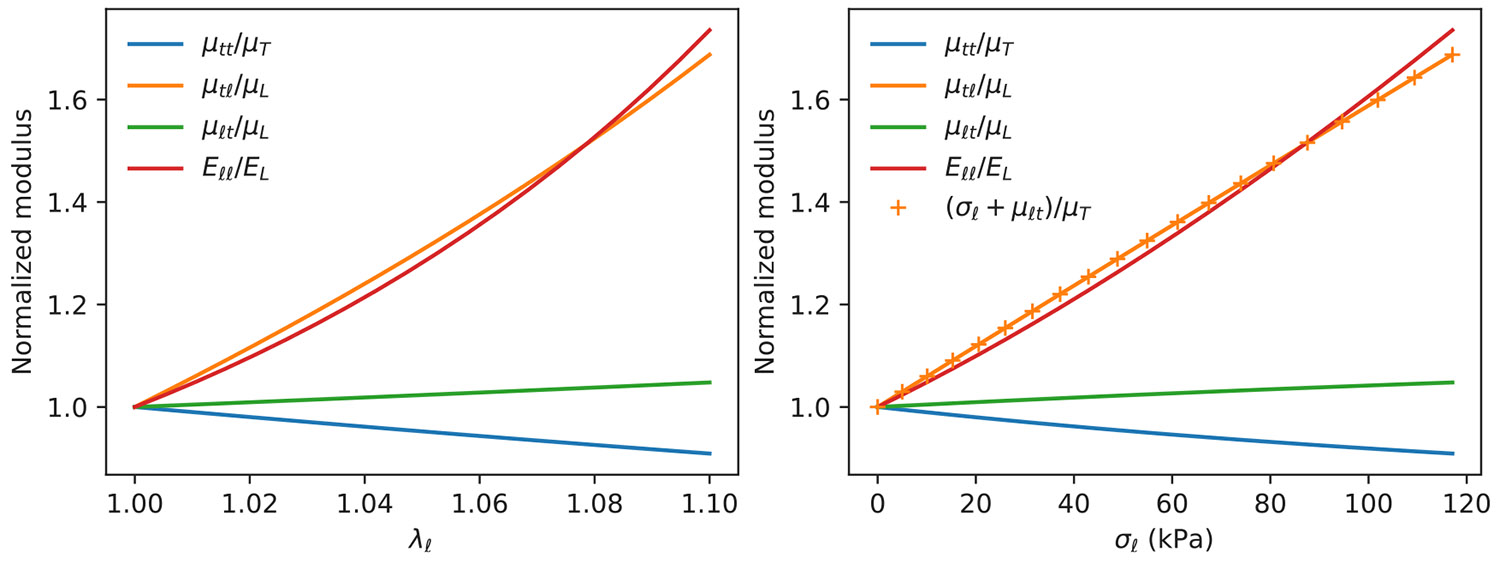
Incremental shear moduli as a function of longitudinal extension, using the parameters in Table 2 and plotted as a function of extension (a) or tensile stress (b).

A wide variety of material properties can be obtained from an experimentally validated strain energy function. These include the incremental moduli, four of which are plotted as a function of the pre-stretch in Fig. 7. The experimentally determined values of the parameters listed in Table 2 where used to generate these curves. The following points are most relevant to our discussion:

- Two of the incremental shear moduli, $\mu_{tt}$ and $\mu_{\ell t}$ depend only weekly on $\lambda_{\ell }$.
- The relatively strong dependence of $\mu_{t\ell }$ on $\lambda_{\ell }$ is due to the effect of the tensile stress on this property, as described by Eq. (15). This fact is made by plotting the incremental moduli as a function of the tensile stress, $\sigma_{\ell }$, as is done in Fig. 7b.
- Strain hardening in extension, quantified by the parameter $c_{4}$ in the strain energy function, gives a noticeable increase in $E_{\ell \ell }$ as the material is extended in the longitudinal direction.

The equations for the relevant incremental moduli are particularly simple in the case where $c_{2}$, the parameter in the model that describes the isotropic strain hardening, can be assumed to be zero. This situation holds in our case, where noticeable strain hardening is observed for extension along the longitudinal axis, but not for extension in the transverse direction (see Fig. 4). In addition to the general relationship connecting $\mu_{t\ell }$ to $\mu_{\ell t}$ and the axial stress (Eq. (15)), we obtain the following expressions when $c_{2}=0$:

(26) $$\mu_{\ell t}=\mu_{t\ell }-\sigma_{\ell }=\frac{\lambda_{\ell }^{2}(\mu_{L}-\mu_{T})+\mu_{T}}{\lambda_{\ell }},$$

(27) $$\mu_{tt}=\frac{\mu_{T}}{\mu_{\ell }}$$

The finite element data used to develop expressions for the contact moduli are based on a linear analysis that does not directly account for strain hardening or a pre-stretch. These equations can still be used to describe indentation of a pre-stretched material if we substitute the different moduli with the appropriate incremental moduli. The substitutions for $E_{L}$ and $\mu_{T}$ are straightforward:

(28) $$E_{L}\to E_{\ell \ell }\;\;\mu_{T}\to \mu_{tt}$$

The substitution for $\mu_{L}$ is not as obvious because the tensile pre-stress affects the parallel shear modulus differently, based on the orientation of the shear displacement with respect to the direction of the applied stress. We can define two limiting contact moduli, $s_{⊥}^{t\ell }$ and $s_{⊥}^{\ell t}$, based on the use of either $\mu_{t\ell }$ or $\mu_{\ell t}$ in place of $\mu_{L}$ in the determination of the contact modulus:

(29) $$s_{⊥}^{t\ell }\;:\mu_{L}\to \mu_{t\ell }\;s_{⊥}^{\ell t}\;:\mu_{L}\to \mu_{\ell t}$$

Values for $s_{∥}^{t\ell }$ and $s_{∥}^{\ell t}$ can be similarly defined, but are nearly identical because $s_{∥}$ has a negligible dependence on $\mu_{L}$.

An understanding of the role of pre-strain on $s_{⊥}$ is complicated by the nonuniform nature of the strain field beneath the indenter, so it is not immediately obvious as to what value we should use for $\mu_{L}$ ($\mu_{t\ell }$, $\mu_{\ell t}$ or some intermediate value) in the linearized analysis. To address this issue for the geometric parameters relevant to our indentation experiments, we calculated the values of $s_{∥}$ and $s_{⊥}$ by finite element analysis, using the full HWM strain energy function (Eq. (11)) and the material parameters listed in Table 2. Details are included in the supporting information. Calculated values of $s_{∥}$ and $s_{⊥}$ from this approach are listed in Table 3 for the four different values of the pre-stretch studied experimentally ($\lambda_{\ell }=1$, 1.026, 1.052, 1.078). The resultant values of $s_{∥}$ and $s_{⊥}$ are shown under the ’FEA with HYM’ heading in Table 3. These values for $s_{⊥}$ were used to normalize the indentation curves of perpendicular indentation shown in Fig. 5(b). As expected, the parallel contact stiffness is independent of the applied stress, but the perpendicular contact stiffness increases as $\lambda_{\ell }$ and the corresponding tensile stress increase. Furthermore, we see that the value of $s_{⊥}$ obtained from the complete FEA solution is in agreement with the value obtained from the linearized FEA model where $\mu_{t\ell }$ is substituted for $\mu_{L}$. Our rationalization of this result is that the dominant shear displacements for indentation in the transverse direction are necessarily in the transverse direction, giving a shear response determined by $\mu_{tl}$.

Finally, we note that the mechanical properties of the gels prepared in this study, shown in Table 2, are indeed representative of skeletal muscle tissues. The moduli of passive muscles are generally lower than the values listed in this table [42]. However, the moduli increase substantially upon activation. Fully activated muscles operating at their optimal length can exhibit a Young’s modulus of approximately 5 MPa, as estimated from reported maximum stress and stiffness values [30, 43]. With $E_{L}\approx 1$ MPa, our gels correspond well to the properties of moderately contracting muscles. Shear wave velocities have also been used to estimate the incremental shear modulus, $\mu_{tl}$, of muscles. Reported velocities of 2–20 m/s, spanning the range from passive to fully activated states [27,44]. correspond to incremental moduli between 4 and 400 kPa — again within the range of our gels. Furthermore, shear wave velocities measured along the longitudinal and transverse directions can be used to evaluate the ratio $\mu_{tl}∕\mu_{tt}$. Gennisson et al. [21] observed a ratio of approximately 4 for moderate contractions, which closely matches the properties of our gels. Overall, with mechanical characteristics representative of moderately contracting muscles, our gels offer a valuable model system for investigating the mechanics of active muscles.

### 4.4. Connection to shear wave elastography

As mentioned in the introduction and referred to in the previous section, sound wave propagation measurements are commonly used to measure elastic constants in soft materials. For this reason we conclude with an extension of our methodology to the calculation of wave speeds in transversely isotropic materials. Our treatment is equivalent to the recent work of Rouze et al. [35] but with the use of the HYM strain energy function and with a notation based on our definitions of the various incremental moduli. These incremental moduli make a direct connection to the phase velocities of shear waves traveling through the material. The phase velocities, for the tt, tℓ and ℓt modes illustrated in Fig. 1, for example, are given by the following expressions:

(30) $$v_{tt}=\sqrt{\frac{\mu_{tt}}{\rho }};\;v_{t\ell }=\sqrt{\frac{\mu_{t\ell }}{\rho }};\;v_{\ell t}=\sqrt{\frac{\mu_{\ell t}}{\rho }}$$

where ρ is the material density. In the context of shear wave propagation, the first letter in the mode label describes the polarization (shear displacement) direction and the second describes the propagation direction. For example, $v_{t\ell }$ is the phase velocity for a wave polarized in the transverse direction propagating in the longitudinal direction.

**Fig. 8.**
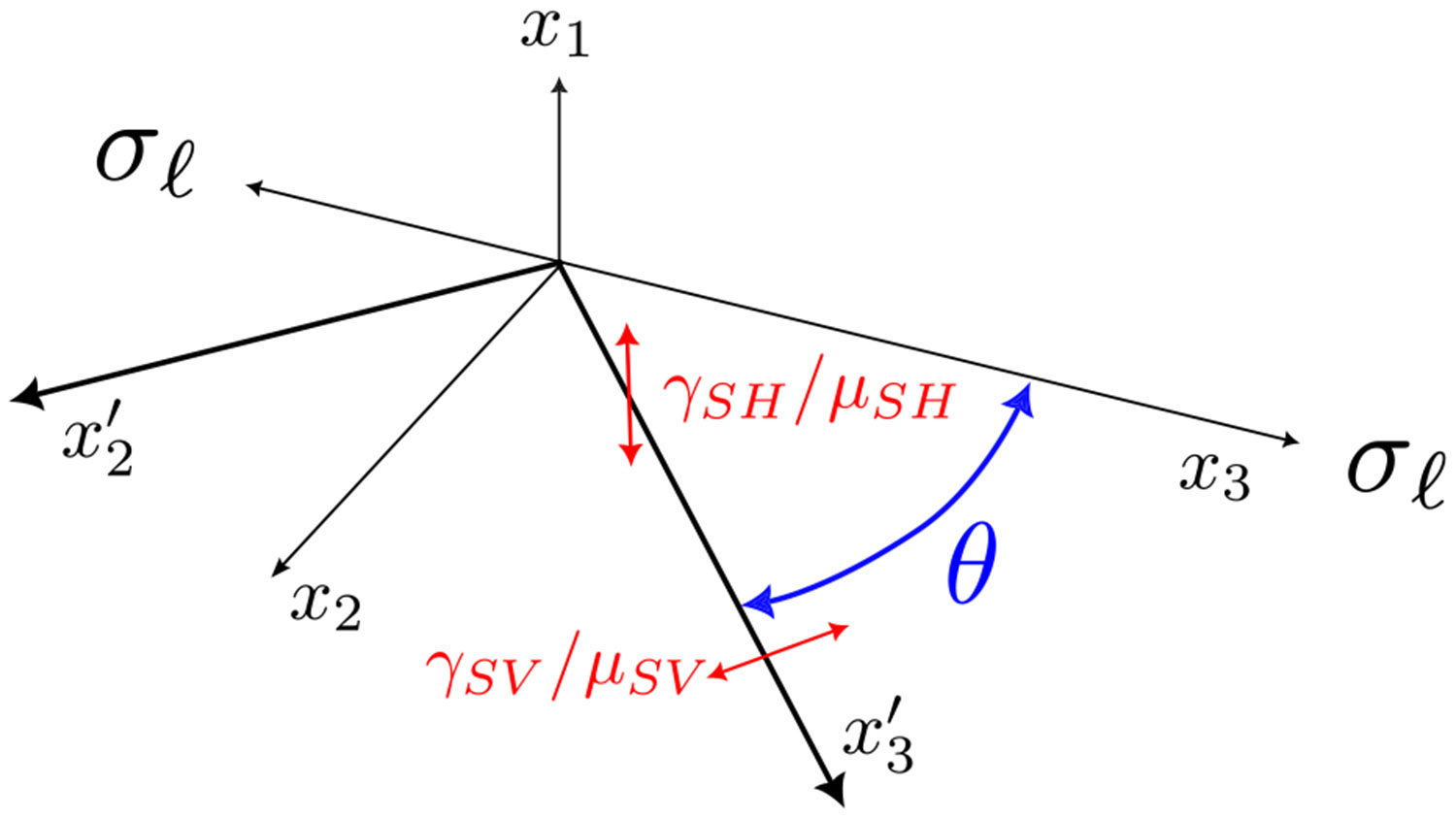
Wave propagation geometry showing the propagation direction and polarization for the shear-horizontal (SH) and shear-vertical (SV) modes. Use of the primed coordinate system obtained by rotating by θ around $x_{1}$ is described in the text.

The situation described above for the highly symmetric modes, with the waves traveling either along the symmetry axis or perpendicular to it, is particularly simple. The approach can be generalized to treat waves traveling in a direction inclined at some angle θ to the symmetry axis as illustrated in Fig. 8. Because the material is isotropic in the $x_{1}-x_{2}$ plane, and the only externally applied stress is a uniaxial stress applied along $x_{3}$, we can define $x_{2}$ so that the propagation direction lies within the $x_{2}-x_{3}$ plane, without any further loss of generality. The relevant plane waves are obtained as the solution to an eigenvalue problem, as described previously [28,34,45,46]. For an incompressible transversely isotropic material two polarizations are obtained: one in the plane containing the propagation direction and the symmetry axis (the $x_{2}-x_{3}$ plane in Fig. 8) and a second in the direction perpendicular to this (the $x_{1}$ direction). Different notations have been used to designate these shear waves in the literature. We use the common convention where the wave polarized along $x_{1}$ is the shear-horizontal (SH) wave and the wave polarized in the $x_{2}-x_{3}$ plane (the plane containing the propagation direction and the symmetry axis) is the shear-vertical (SV) wave [26,35,47,48]. Moduli corresponding to these two propagation modes are related to the corresponding phase velocities through equations analogous to Eq. (30):

(31) $$v_{SH}(\theta )=\sqrt{\frac{\mu_{SH}(\theta )}{\rho }};\;v_{SV}(\theta )=\sqrt{\frac{\mu_{SV}(\theta )}{\rho }}$$

For waves traveling along the symmetry axis (θ=0) the SH and SV modes are equivalent, with $\mu_{SV}=\mu_{SH}=\mu_{t\ell }$. For waves traveling in the transverse direction (θ=90°) we have $\mu_{SV}=\mu_{\ell t}$ and $\mu_{SH}=\mu_{tt}$. Transversely isotropic materials investigated by shear wave elastography (most notably skeletal muscle) have $\mu_{L}>\mu_{T}$, and hence $\mu_{\ell t}>\mu_{tt}$ and $v_{SV}>v_{SH}$. For this reason the SV and SH modes are often referred to as the ‘fast’ and ‘slow’ modes, respectively, although this relative relationship between the wave speeds is not necessarily valid for all transversely isotropic materials.

Calculation of $\mu_{SH}(\theta )$ and $\mu_{SV}(\theta )$ proceeds in the same way as for the calculation of the other incremental moduli from Eqs. (12) and (13), but with a more generalizable form. We begin by defining a new, primed coordinate system rotated by θ around the $x_{1}$ axis. The original coordinate sytem has $x_{3}$ oriented along the symmetry axis of the fiber, whereas this primed coordinate system has $x_{3}^{\prime }$ oriented along the direction of travel for the shear wave of interest. The incremental deformation gradient characterizing the shear deformation in this primed coordinate system is:

(32) $$F_{inc}^{\prime }=\left[\begin{array}{ccc} 1 & 0 & \gamma_{SH}\\ 0 & 1 & \gamma_{SV}\\ 0 & 0 & 1 \end{array}\right]$$

Here $\gamma_{SV}=0$ for the SH mode and $\gamma_{SH}=0$ for the SV mode. We obtain $F_{inc}$ in the original coordinate system by applying the matrix transformation for a rotation by θ about the $x_{1}$ axis:

(33) $$F_{inc}={\mathrm{SF}}_{inc}^{\prime }S^{T};S=\left[\begin{array}{ccc} 1 & 0 & 0\\ 0 & \cos\;\theta & -\sin\;\theta \\ 0 & \sin\;\theta & \cos\;\theta \end{array}\right]$$

Use of this value for $F_{inc}$ ensures that the calculations for the stress are done in the original coordinate frame with the symmetry axis of the material aligned along the $x_{3}$ direction. The stress obtained from Eq. (12) needs to be transformed back to the primed coordinate system by the appropriate rotation by θ:

(34) $$\sigma^{\prime }=S^{T}\sigma S$$

As in Eq. (13), values of $\mu_{SH}$ and $\mu_{SV}$ are obtained by differentiation of the appropriate stress component with respect to the incremental shear strain:

(35) $$\mu_{SH}(\lambda_{\ell })=\frac{\partial \sigma_{13}^{\prime }\;(\lambda_{\ell },\gamma_{SH})}{\partial \gamma_{SH}};\;\mu_{SV}(\lambda_{\ell })=\frac{\partial \sigma_{23}^{\prime }\;(\lambda_{\ell },\gamma_{SV})}{\partial \gamma_{SV}}$$

**Fig. 9.**
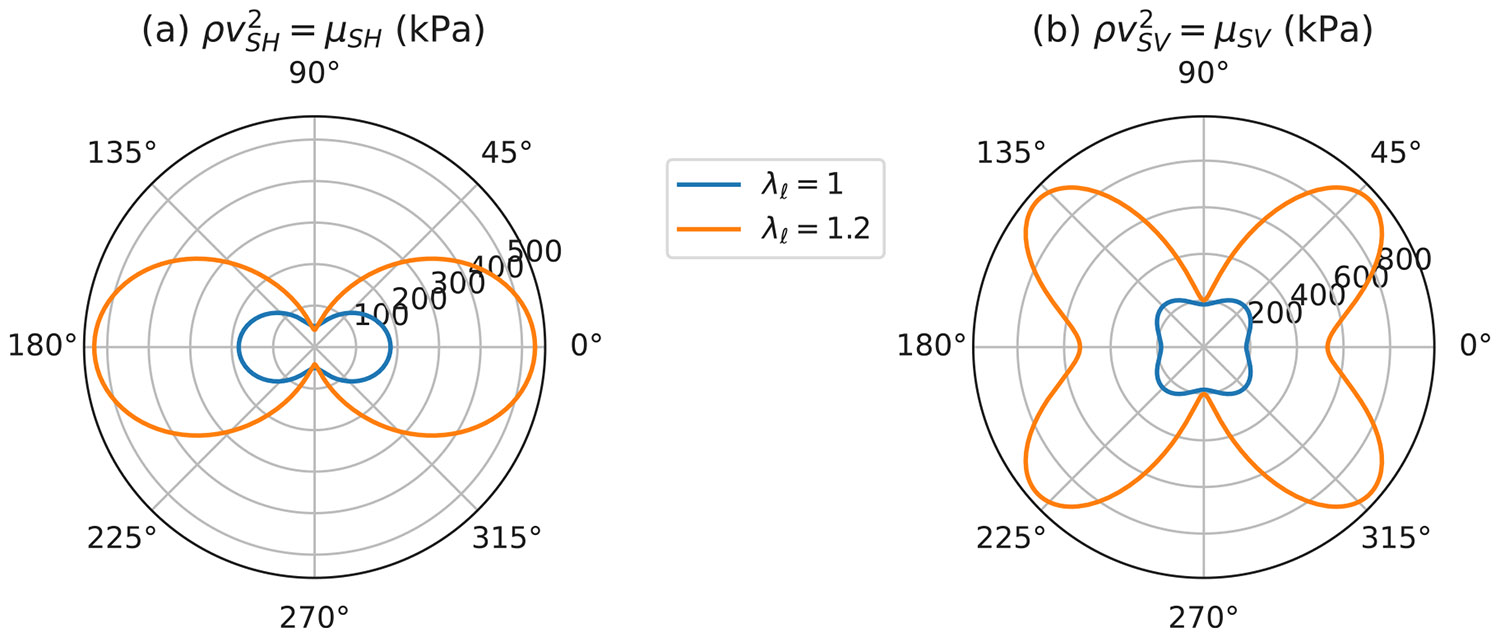
Dependence on the orientation angle θ on the incremental moduli characterizing the SH mode (a) and the SV mode (b) for $\lambda_{\ell }=1$ and $\lambda_{\ell }=1.2$ 8.

Polar plots of $\mu_{SH}$ and $\mu_{SV}$ as a function of θ are shown in Fig. 9 for $\lambda_{\ell }=1$ and 1.2, using the materials parameters listed in Table 2.

Ultrasound shear wave elastography typically involves measurements of the propagation velocity for a wave that initiates from a pulse excited within the material. The relevant propagation velocities in this case are the group velocities, V, and not the phase velocities given by Eq. (31). Fortunately, the phase and group velocities are related to each other in a straightforward way [35]. With our definitions, where the symmetry axis is aligned along $x_{3}$ and where the wave propagates in the $x_{2}-x_{3}$ plane, we have the following for the 2 and 3 components of the group velocity:

(36) $$V_{2}=v\;\sin(\theta )+\frac{\partial v}{\partial \theta }\;\cos\;\theta \;V_{3}=v\;\cos(\theta )-\frac{\partial v}{\partial \theta }\;\sin\;\theta$$

The overall group velocity is given by:

(37) $$V=\sqrt{V_{2}^{2}+V_{3}^{2}}$$

This group velocity corresponds to waves traveling in a direction inclined to the symmetry axis by an angle $\theta_{g}$, with $\theta_{g}$ given by:

(38) $$\theta_{g}=\theta +\arctan\left(\frac{1}{v}\frac{\partial v}{\partial \theta }\right)$$

**Fig. 10.**
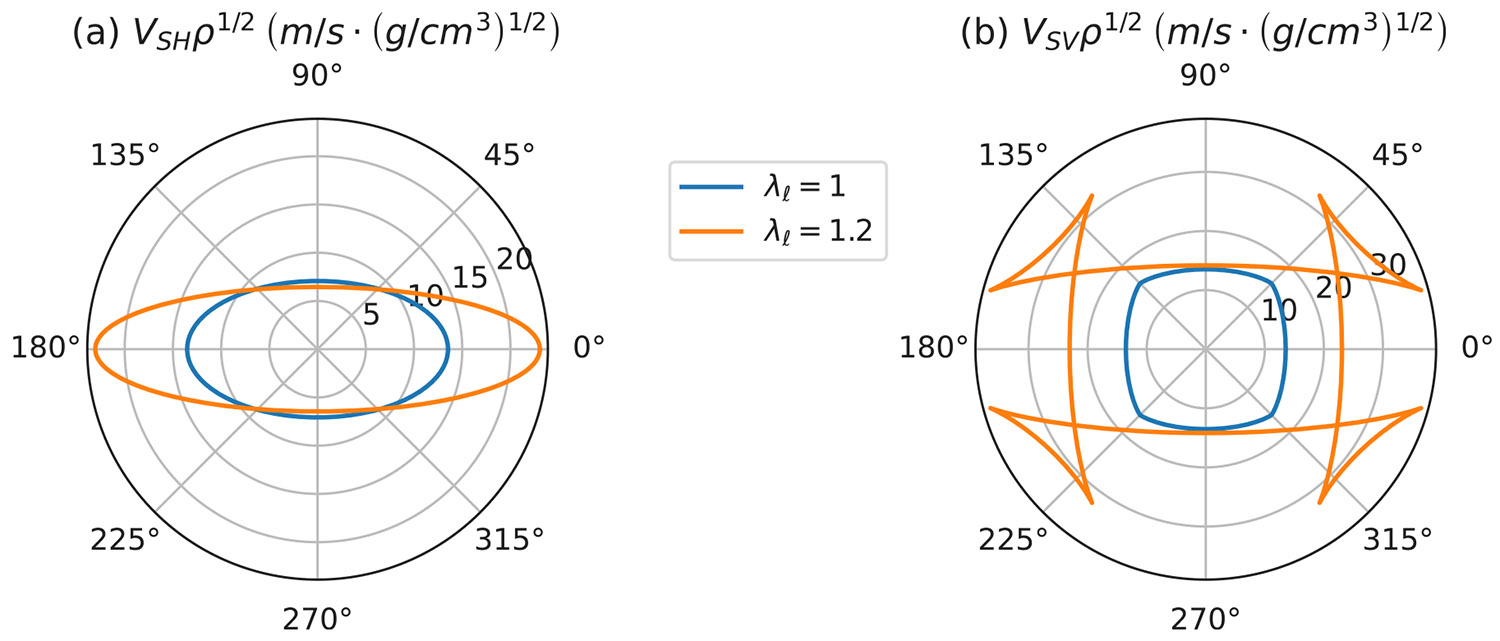
Dependence on the orientation angle $\theta_{g}$ of the group velocities for the SV and SH waves for the data from Fig. 9.

These equations hold independently for v, $V=v_{SH}$, $V_{SH}$ and v, $V=v_{SV}$, $V_{SV}$. Polar plots of both group velocities as a function of the propagation direction, $\theta_{g}$ for a wavefront are illustrated in Fig. 10 for the same set of parameters used to generate the plots in Fig. 9. Note that the velocities are always multiplied by the square root of the density. We use g/cm^3^ as the density unit, giving a density close to one for our materials, with predicted group velocities in m/s that are easily determined from the plots.

#### 4.4.1. Expressions for the stress-free limit

Our approach is to use the symbolic math capabilities of Python to carry out the matrix transformations and differentiations that are required to obtain the relevant moduli. The equations are solved exactly and stored in as analytic functions that enable the stresses and moduli to be obtained directly for specified material parameters. In most cases these functions are much too lengthy to be written down. There are some exceptions, however, where relatively simple results are obtained. An important example includes the stress-free limit ($\lambda_{\ell }=1$) where the following expressions are obtained:

(39) $$\mu_{SH}(\theta ,\;\lambda_{\ell }=1)=\mu_{L}\;\cos^{2}\;\theta +\mu_{T}\;\sin^{2}\;\theta$$

(40) $$\mu_{SV}(\theta ,\;\lambda_{\ell }=1)=\mu_{L}\;\sin^{2}\;\theta \;\cos^{2}\;\theta (E_{L}+\mu_{T}-4\mu_{L})$$

For the group velocity for the shear-horizontal mode we obtain:

(41) $$\rho V_{SH}^{2}(\theta )=\frac{\mu_{L}\mu_{T}}{\mu_{L}\;\sin^{2}\;(\theta_{g})+\mu_{T}\;\cos^{2}\;(\theta_{g})}$$

These expressions are identical to those given previously for an unstressed, transversely isotropic material [47,49,50].

#### 4.4.2. Expression for the SH mode with tensile stress

The modulus characterizing the SV mode in the presence of an applied tensile stress along the symmetry axis is quite complicated, and is not discussed in more detail here. The situation for the SH mode is simpler, however, because in this case the polarization is perpendicular to the symmetry axis for all values of θ. This situation enables us to substitute $\mu_{t\ell }$ for $\mu_{L}$ and $\mu_{tt}$ for $\mu_{T}$ in Eq. (39):

(42) $$\rho v_{SH}^{2}(\theta )=\mu_{SH}(\theta )=\mu_{t\ell }\;\cos^{2}\;\theta +\mu_{tt}\;\sin^{2}\;\theta =(\mu_{\ell t}+\sigma_{\ell })\;\cos^{2}\;\theta +\mu_{tt}\;\sin^{2}\;\theta$$

A corresponding substitution can be made or the expression for the group velocity, so we obtain:

(43) $$\rho V_{SH}^{2}(\theta )=\frac{\mu_{t\ell }\mu_{tt}}{\mu_{t\ell }\;\sin^{2}\;(\theta_{g})+\mu_{tt}\;\cos^{2}\;(\theta_{g})}=\frac{(\mu_{\ell t}+\sigma_{\ell })\;\mu_{tt}}{\left(\mu_{\ell t}+\sigma_{\ell })\;\sin^{2}\;(\theta_{g})+\mu_{tt}\;\cos^{2}\;(\theta_{g}\right)}$$

The forms of these expressions involving $\sigma_{\ell }$ are useful because they more clearly illustrate the role of the stress in the wave speeds, since $\mu_{\ell t}$ and $\mu_{tt}$ are only weakly dependent on the deformation. To illustrate further, we consider the case where $c_{2}=0$, where we can use Eqs. (26) and (27) for $\mu_{\ell t}$ and $\mu_{tt}$, respectively, to obtain the following:

(44) $$\mu_{SH}(\theta )=\left(\frac{\lambda_{\ell }^{2}\left(\mu_{L}-\mu_{T}\right)+\mu_{T}}{\lambda_{\ell }}+\sigma_{\ell }\right)\cos^{2}\theta +\frac{\mu_{T}}{\lambda_{\ell }}\;\sin^{2}\;\theta$$

## 5. Summary

In this work we have used the HYM strain energy function developed by Hegde et al. as a basis for understanding the elastic response of an incompressible transversely isotropic material. This strain energy function is written in terms of the three independent elastic constants that are minimally required to specify the linear elastic response ($E_{L}$, $\mu_{L}$, $\mu_{T}$) in addition to two additional parameters ($c_{2}$, $c_{4}$) to quantify the strain hardening. The direct physical meaning of the parameters appearing in the HYM strain energy function, in addition to its relative simplicity, motivate our decision to use it in our work. While $c_{2}$ and $c_{4}$ must necessarily be non-negative to describe the material at sufficiently large strains, a small negative value of $c_{2}$ was found to improve the ability of the HYM function to completely describe the measured extensional properties of the material in the transverse direction at intermediate strains. Nevertheless, strain hardening in the direction of the symmetry axis dominates the behavior of our model gels, as we expect to be the case for many other materials of interest as well. In these cases it is sufficient to take $c_{2}=0$, so the material response is now specified by 4 independent parameters. We used a combination of uniaxial extension and indentation with a high aspect ratio, rectangular indenter to assess the properties of a model soft, transversely isotropic material. Our primary results can be summarized as follows:

- Since only two elastic constants can be obtained from the indentation measurements, at least one must be obtained from an additional mechanical measurement. In our this elastic constant is $E_{L}$, determined from uniaxial extension in the direction of the symmetry axis. We expect this to be the most obvious choice in most other applications as well. The strain hardening coefficient, $c_{4}$ is obtained from this same measurement.
- The easiest elastic constant to obtain from an indentation measurement is the transverse shear modulus, $\mu_{T}$, obtained from a contact measurement where the long axis of the indenter is aligned along the symmetry axis of the material.
- Once $E_{L}$ and $\mu_{T}$ are known, $\mu_{L}$ is obtained from a contact measurement where the long axis of the indenter is aligned in the direction perpendicular to the symmetry axis of the material. Data needed to extract the moduli from the contact measurements are provided, and enable others to perform this analysis without repeating the finite element investigations performed here.
- For a material being stressed uniaxially along the symmetry axis, the moduli obtained from the indentation measurements are obtained by replacing $E_{L}$, $\mu_{T}$, $\mu_{L}$ with the respective incremental moduli, $E_{\ell \ell }$, $\mu_{tt}$ and $\mu_{t\ell }$. The validity of these substitutions was verified with finite element analysis simulations of indentation on prestressed materials using the full HYM strain energy function.
- The definitions of the incremental moduli are also in helping to understand ways that the elastic constants can be used to determine both the tensile stress and the elastic constants from shear wave elastography. The approach was illustrated by using the HYM and function to determine the phase and group velocities or the model gel used in our experiments.

## Supplementary Material

Supporting Information

## Figures and Tables

**Table 1:** Relationships between the different elastic constants for an incompressible material with fiber (orthotropic) symmetry.

	$\mu_{T}$	$E_{T}$	$E_{L}$	$\nu_{tt}$
$\mu_{T}$, $E_{T}$	–	–	$\frac{E_{T}\mu_{T}}{4\mu_{T}-E_{T}}$	$\frac{E_{T}}{2\mu_{T}}-1$
$\mu_{T}$, $E_{L}$	–	$\frac{4E_{L}\mu_{T}}{E_{L}+\mu_{T}}$	–	$\frac{E_{L}-\mu_{T}}{E_{L}+\mu_{T}}$
$\mu_{T}$, $\nu_{tt}$	–	$2\mu_{T}(\nu_{tt}+1)$	$\frac{\mu_{T}(1+\nu_{tt})}{1-\nu_{tt}}$	–
$E_{T}$, $E_{L}$	$\frac{E_{T}E_{L}}{4E_{L}-E_{T}}$	–	–	$1-\frac{E_{T}}{2E_{L}}$
$E_{T}$, $\nu_{tt}$	$\frac{E_{T}}{2(1+\nu_{tt})}$	–	$\frac{E_{T}}{2(1-\nu_{tt})}$	–
$E_{L}$, $\nu_{tt}$	$\frac{E_{L}\cdot (1-\nu_{tt})}{1+\nu_{tt}}$	$2E_{L}\cdot (1-\nu_{tt})$	–	–

**Table 2:** Elastic constants used to fit indentation and extension data for the model transversely isotropic gels. The errors in the values of $E_{L}$, $\mu_{T}$ and $\mu_{L}$ are from the procedure outlined in the text for obtaining these three parameters from a combination of longitudinal extension and blade indentation with the long axis of the blade oriented in the longitudinal and transverse directions.

$E_{L}$ (kPa)	$\mu_{T}$ (kPa)		$\mu_{L}$ (kPa)	$c_{2}$	$c_{4}$
	Tensile	indentation			
942 ± 47	49.7 ± 2.6	51.0 ± 5	183 ± 70	−0.43	18.6

**Table 3:** Calculated incremental moduli and contact stiffness for different values of the pre-stretch, using the parameters from Table 2.

$\lambda_{\ell }$	From HYM function Eq. (11)					FEA with HYM		FEA with linear model			
	$\sigma_{\ell }$ (kPa)	$E_{\ell \ell }$ (kPa)	$\mu_{tt}$ (kPa)	$\mu_{\ell t}$ (kPa)	$\mu_{t\ell }$ (kPa)	^a^ $s_{∥}$ (kPa)	^a^ $s_{⊥}$ (kPa)	^b^ $s_{∥}^{\ell t}$ (kPa)	^b^ $s_{⊥}^{\ell t}$ (kPa)	^c^ $s_{∥}^{t\ell }$ (kPa)	^c^ $s_{⊥}^{t\ell }$ (kPa)
1	0	942	49.7	183	183	51.8	190	50.8	187	50.8	187
1.026	25.7	1065	48.4	185	211	51.9	210	49.8	200	49.9	209
1.052	56.5	1221	47.1	187	242	52.0	234	48.7	216	49.0	235
1.078	86.3	1421	45.7	189	276	52.0	262	47.7	235	48.1	265

a $s_{∥}$ and $s_{⊥}$ from hyperelastic model defined as function Eq. (11).

b $s_{∥}$ and $s_{⊥}$ from prestress free linear elastic model with $E_{\ell \ell }$, $\mu_{tt}$ and $\mu_{\ell t}$ as input.

c $s_{∥}$ and $s_{⊥}$ from prestress free linear elastic model with $E_{\ell \ell }$, $\mu_{tt}$ and $\mu_{t\ell }$ as input.

## Appendix A. Supplementary data

Supplementary material related to this article can be found online at https://doi.org/10.1016/j.actbio.2025.10.062. Supporting information includes background on the elastic constants for a transversely tropic material and relationships between them, details of the experimental and finite element modeling procedures, and Python scripts used to generate figures in the paper.
