# Supplementary material for: Mechanical characterization of an incompressible, strain-hardening, transversely isotropic material
Source: Acta Biomater. Author manuscript; Available in PMC 2026 May 2. (PMC13135306; doi:10.1016/j.actbio.2025.10.062)
Supplement: Supporting Information [file NIHMS2166262-supplement-Supporting_Information.pdf]

Supporting Information

Mechanical Characterization of an Incompressible, Strain-Hardening, Transversely Isotropic Material

Qifeng Wang[a], Sheng Wang[a,1], Mahdi Ebrahimkhani[b,c], Thomas J. Royston[d], Eric J. Perreault[b,c,e], Kenneth R. Shull[a,]

[a]*Department of Materials Science and Engineering, Northwestern University, 2220 Campus Drive, Evanston, 60208, IL, USA*
[b]*Department of Biomedical Engineering, Northwestern University, 2145 Sheridan Road, Evanston, 60208, IL, USA*
[c]*Shirley Ryan AbilityLab, 355 E Erie Street, Chicago, 60611, IL, USA*
[d]*Richard and Loan Hill Department of Biomedical Engineering, University of Illinois Chicago, 851 South Morgan Street, Chicago, 60607, IL, USA*
[e]*Department of Physical Medicine and Rehabilitation, Northwestern University, 710 N. Lake Shore Drive, Chicago, 60611, IL, USA*

Contents

1 Experimental Details **2**
1.1 Instruments . . . 2
1.2 Gel Preparation . . . 2
1.3 Tensile testing . . . 2
1.4 Microindentation testing . . . 2

2 Elastic Constants of an Incompressible, Transversely Isotropic Material **3**

3 Finite Element Analysis **3**
3.1 General Considerations . . . 3
3.2 Hyperelastic Model . . . 4
3.3 Determination of the a/horrection for Parallel Indentation . . . 5

4 Analytic Expressions for Various Quantities **6**
4.1 True stress for longitudinal extension. . . . 6
4.2 μ_{tt} . . . 6
4.3 $\mu_{\ell t}$ . . . 6
4.4 $\mu_{t\ell}$ . . . 6
4.5 μ_{SH} . . . 6
4.6 μ_{SV} . . . 6

5 Python Code Used to Generate Figures **7**
5.1 Figure 4 . . . 7
5.2 Figure 5 . . . 8
5.3 Figure 6 . . . 11
5.4 Figure 7 . . . 12
5.5 Figures 9 and 10 . . . 13
5.6 Functions for Elastic Analysis (swe.py) . . . 15

[1]Current location: Department of Materials Science and Engineering, University of Wisconsin-Madison, Madison, WI 53706, United States

1. Experimental Details

1.1. Instruments

A rheometer (Anton Paar MCR302) with a concentric cylinder adapter (cc27, 1.13 mm gap) was used for the preparation of transversely isotropic gels and *in-situ* rheology measurements of as-prepared gels. DMA (TA Instruments RSA3) was used for tensile tests to measure the Young's moduli of as-prepared gels. Asymmetric micro-indentation tests were carried out on a lab-made apparatus described in the previous work.[1] The stainless-steel indenter has a contact cross-section of 0.14 mm in width, 20 mm in length, and the ends along the longer axis are rounded with 0.3 mm radius.

1.2. Gel Preparation

The transversely isotropic gels were prepared on the rheometer by applying a constant shear rate. PMMA-PnBA-PMMA triblock copolymer was dissolved in 2-ethyl-hexanol with 22 wt.% PMMA in the solution. The solution was preheated to about 90 °C under stirring to completely dissolve of the copolymer. Then the solution was kept at the same temperature without stirring for 3 to 6 hours to eliminate air trapped in the solution. The hot solution without bubbles was carefully transferred to the preheated (90 °C) concentric cylinder adapter on the rheometer. An extra 30 min to 60 min was added before the next step to let the air bubbles come out, which were introduced while transferring. The solution was then cooled to60 °C and kept at that temperature for 10 min with a constant shear rate of 0.15 1/s for the formation and alignment of cylindrical micelles. Then the shear was turned off and the temperature was set to 25 °C to reach the maximum cooling (about 5 °C/min) to immobilize the alignment of the micelles by solidifying the solution to a gel. The formed gel was carefully taken out of the adapter and only the thin sheet (about 1.1 mm thick) on the side was used for the mechanical tests. To ensure consistent properties across all samples, a single gel sheet was used as the source material.

1.3. Tensile testing

Tensile tests were performed on the as-prepared single sheet (1.1 mm thick) which was cut to a length of 15-20 mm long and about 5 mm wide with longer edge in either the longitudinal direction (axis 3) or the transverse direction (axis 2). The samples were mounted on the DMA with an active lengths about 10 mm and were stretched along their longer edge at a speed of 0.1 mm/s.

1.4. Microindentation testing

As-prepared sheets gel sheets cut to about 25 mm (axis 2) and 30-40 mm (axis 3) were used for the asymmetric indentation tests. The two end edges (25 mm long, perpendicular to the fiber) of the thin sample were glued onto a 3D-printed fixture connected with a micrometer. Therefore, the extension of the gels along the longitudinal direction (axis 3) is quantitatively controlled. The rectangular indenter was brought to contact with the sample at a speed of 0.01 mm/s until the desired load and was brought back away from the sample. Indentation tests with the long edge of the indenter parallel and perpendicular to the symmetry axis of the gels were tested with the samples being stretched to different extensions in the longitudinal direction.

To confirm the validity of the transversely isotropic properties of the thin gel sheets, thicker samples with X_1=12.5 mm , X_2 = 14.0 mm , X_3= 13.8 mm were prepared by welding the thin sheets (1.1 mm) together by stacking multiple thin sheets under a light

pressure at 55 °C, which is 5 °C below the critical micelle temperature this polymer, for 30 minutes. Symmetric indentations with a cylindrical indenter were performed on directions 1 and 2 on these welded samples. These experiments showed that the properties in these two directions are identical within experimental error.

2. Elastic Constants of an Incompressible, Transversely Isotropic Material

The stiffness matrix for a transversely isotropic material (not necessarily incompressible) can be written in the following way:[2]

$$C = \begin{bmatrix} K_T + \mu_T & K_T - \mu_T & 2K_T\nu_{\ell t} & 0 & 0 & 0 \\ K_T - \mu_T & K_T + \mu_T & 2K_T\nu_{\ell t} & 0 & 0 & 0 \\ 2K_T\nu_{\ell t} & 2K_T\nu_{\ell t} & E_L + 4K_T\nu_{\ell t}^2 & 0 & 0 & 0 \\ 0 & 0 & 0 & \mu_L & 0 & 0 \\ 0 & 0 & 0 & 0 & \mu_L & 0 \\ 0 & 0 & 0 & 0 & 0 & \mu_T \end{bmatrix} \tag{S1}$$

Here K_T, is inversely related to the compressibility of the material. For an incompressible material $\nu_{\ell t} = 0.5$ and $K_T = \infty$. In this case, only three elastic constants are independent of one another, with the relationships described in Table 1.

3. Finite Element Analysis

3.1. General Considerations

COMSOL Multiphysics Version 6.2 (COMSOL, Burlington, MA) software was used for conducting the numerical FE simulations. The model was built with a quarter of the 3D geometry of both the gel and indenter following the symmetric of the system. The indenter material was set to steel AISI 4340. The gel material was defined by the stiffness matrix shown in Eq. S1 with $\nu_{\ell t}$=0.4999 and $\nu_{tt} = \frac{E_L - \mu_T}{E_L + \mu_T}$ to represent its nearly incompressible nature. The full stiffness matrix utilized in the linear simulations is then obtained from the specification of E_L, μ_T and μ_L. Stationary finite element simulations were performed with the long axis of the indenter both parallel and perpendicular to the longitudinal symmetry axis of the material. The meshes were calculated separately for the two different conformations. The mesh of the indenter was a sweep of a free-quad mesh. A finer free-tetrahedral mesh was applied to the gel where it contacts with the indenter, with a minimum element size equal to $a/4$. A maximum element growth rate of 1.3 was used to generate the rest of the gel mesh. Further decreasing the mesh size was found to have a less than 1% change on the properties while prolonging the computing time. An image illustrating the mesh is shown in Figure S1 and the mesh element statistics are shown in Table S1. Since the parallel and perpendicular indentation conditions have only small differences, only the perpendicular condition for both the examples of mesh illustration and statistics are shown. Frictionless boundaries were assumed at the indenter/gel and gel/substrate interfaces. This frictionless boundary condition simplifies the model[3, 4] and is consistent with most use cases, including our model gels that have a relatively high solvent content.

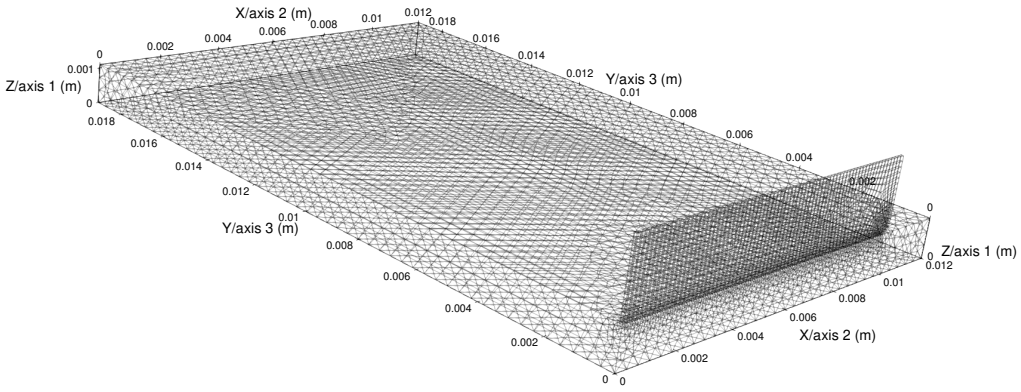

Figure S1: Meshes for the FEA with indenter longer edge perpendicular to the axis 3. The meshes for 90°were generated with the same parameters.

Table S1: Example of the mesh element statistics for perpendicular indentation

Number of elements	48859
Minimum element quality	0.2018
Average element quality	0.667
Element volume ratio	2.387×10^{-5}
Mesh volume	2.126×10^{-7} m^3

3.2. Hyperelastic Model

A user defined hyperelastic model was also used in order to validate our use of the incremental moduli in the linear model. This model utilizes the strain energy function defined by Eq. 11 in the main paper. Simulation results for uniaxial stretching of the gel in the longitudinal direction are in quantitative agreement with the analytic expression for the stress, as illustrated in Figure S2.

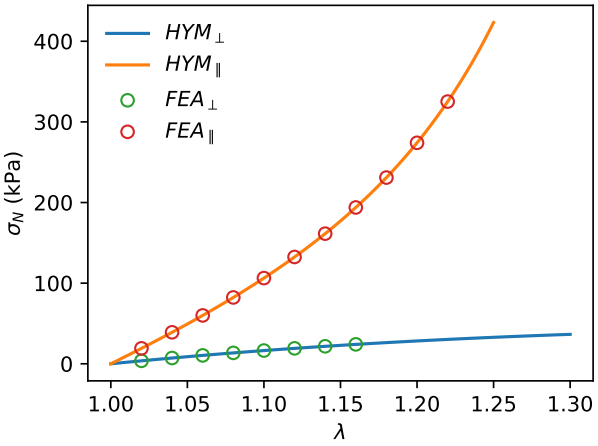

Figure S2: Comparison of solutions from HYM function with the parameters from Table 2 in the main paper (solid lines) and FEA solutions (circle symbols).

The same hyperelastic model was used to determine the contact moduli for the pre-stretched materials. The contact moduli were obtained as the slope of the plot of the

indentation stress as a function of δ/a. The value of $s_\perp$ increases with indentation depth, whereas $s_\parallel$ is independent of the indentation depth as illustrated in Figure S3. The contact moduli at normalized displacement δ/a close to 0 (δ/a=0.02) are listed in Table 3 to compare with FEA results obtained with the linear elastic model.

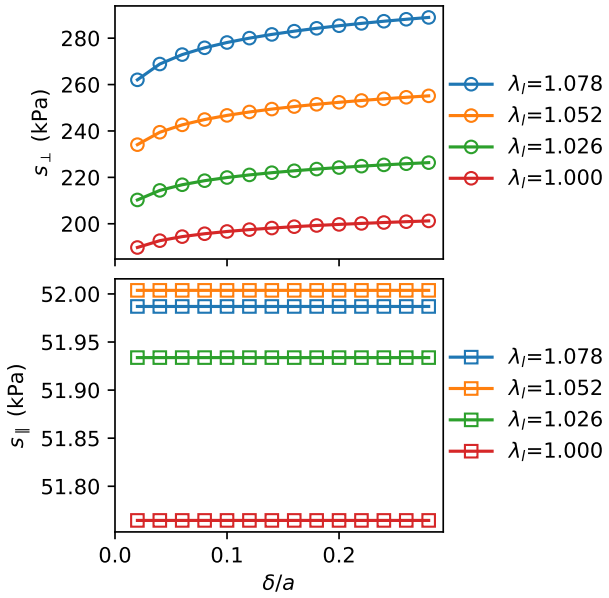

Figure S3: Contact moduli $s_\perp$ and $s_\parallel$ change with normalized indentation displacement d/a.

3.3. Determination of the a/horrection for Parallel Indentation

Values of $\mu_T/s_\parallel$ are plotted as a function of a/h in Figure S4. These data include values of $E_L/(3\mu_T)$ ranging from 1 to 100 and values of μ_L/μ_T ranging from 1 to 100.

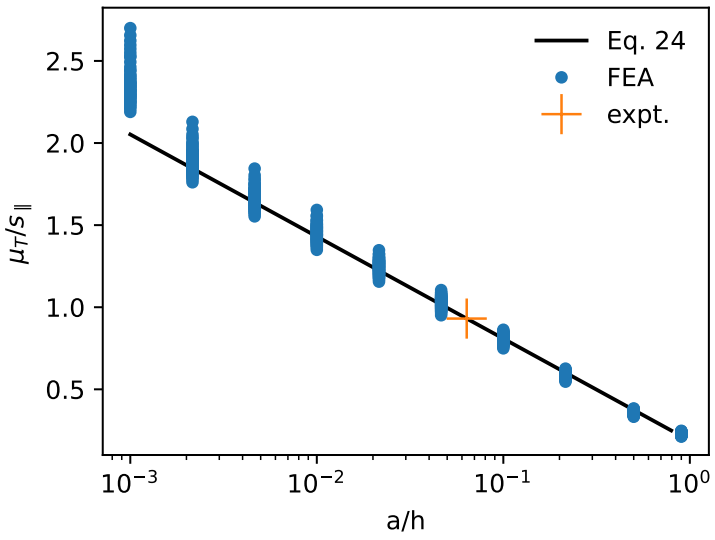

Figure S4: Representation of Eq. 24 (solid line) and FEA simulated data for ℓ/a=400. The different symbols for a given value of a/h correspond to different values of the two material asymmetry parameters, μ_L/μ_T and $E_L/(3\mu_T)$ which both span a range of 1 to 100. The cross represents the experimental data point for the model gel, with $a/h = 0.964$.

4. Analytic Expressions for Various Quantities

For completeness we include analytic expressions for the stress in uniaxial extension and for various incremental moduli defined in the main paper. These are vector graphics images that can be scaled for legibility (particularly important for μ_{SV}).

4.1. True stress for longitudinal extension.

$$\frac{\lambda_\ell^2\left(2\lambda_\ell^3\left(\mu_L-\mu_T\right)+\lambda_\ell\mu_T e^{\frac{c_2\left(\lambda_\ell\left(\lambda_\ell^2-3\right)+2\right)}{\lambda_\ell}}+2\lambda_\ell\left(-\mu_L+\mu_T\right)+\left(\lambda_\ell-1\right)\left(E_L-4\mu_L+\mu_T\right)e^{c_4\left(\lambda_\ell-1\right)^2}\right)-\mu_T e^{\frac{c_2\left(\lambda_\ell\left(\lambda_\ell^2-3\right)+2\right)}{\lambda_\ell}}}{\lambda_\ell}$$

4.2. μ_{tt}

$$\frac{\mu_T e^{\frac{c_2\left(\lambda_\ell\left(\lambda_\ell^2-3\right)+2\right)}{\lambda_\ell}}}{\lambda_\ell}$$

4.3. $\mu_{\ell t}$

$$\frac{\lambda_\ell^2\left(\mu_L-\mu_T\right)+\mu_T e^{\frac{c_2\left(\lambda_\ell\left(\lambda_\ell^2-3\right)+2\right)}{\lambda_\ell}}}{\lambda_\ell}$$

4.4. $\mu_{t\ell}$

$$\lambda_\ell\left(\lambda_\ell\mu_T e^{\frac{c_2\left(\lambda_\ell\left(\lambda_\ell^2-3\right)+2\right)}{\lambda_\ell}}-2\lambda_\ell\left(\mu_L-\mu_T\right)+\left(\lambda_\ell-1\right)\left(E_L-4\mu_L+\mu_T\right)e^{c_4\left(\lambda_\ell-1\right)^2}+\left(2\lambda_\ell^3+1\right)\left(\mu_L-\mu_T\right)\right)$$

4.5. μ_{SH}

$$\frac{\lambda_\ell^2\left(\lambda_\ell\mu_T e^{\frac{c_2\left(\lambda_\ell\left(\lambda_\ell^2-3\right)+2\right)}{\lambda_\ell}}-2\lambda_\ell\left(\mu_L-\mu_T\right)+\left(\lambda_\ell-1\right)\left(E_L-4\mu_L+\mu_T\right)e^{c_4\left(\lambda_\ell-1\right)^2}+\left(2\lambda_\ell^3+1\right)\left(\mu_L-\mu_T\right)\right)\cos^2\left(\theta\right)+\mu_T e^{\frac{c_2\left(\lambda_\ell\left(\lambda_\ell^2-3\right)+2\right)}{\lambda_\ell}}\sin^2\left(\theta\right)}{\lambda_\ell}$$

4.6. μ_{SV}

The equations in this section were generated from the following Python script, which also generates the values of the incremental moduli and stress values listed in Table 2. This script calls functions defined in swe.py, which is also included below.

```python
#equations.py
import swe
import numpy as np
parms = swe.parms
# verfy equation for stress in terms of difference between shear moduli
stress=swe.stress((2,2))[2,2].subs(swe.delta,1)
mu_lt = swe.modulus((2,1))
mu_tl = swe.modulus((1,2))
mu_tt = swe.modulus((0,1))
E_l = swe.modulus((2,2))
diff = swe.simplify(mu_tl-mu_lt-stress)
print(f'diff = {diff}')  # diff=0, so Eq. 15 is valid
lamlvals = np.array([1, 1.026, 1.052, 1.078])

for mod, modname in zip([mu_lt, mu_tl, mu_tt, E_l, stress],
```

```
                        ['mu_lt', 'mu_tl', 'mu_tt', 'E_l', 'stress']):
    modfunc = swe.make_plotable(mod, [swe.laml], parms)
    print(modname)
    modvals = modfunc(lamlvals)
    for i in np.arange(len(lamlvals)):
        print(f'{modvals[i]:.3g}')


#%% calculate the modulie for the SHand SV modes (takes a while)
mu_SH = swe.modulus((0,2,1))
mu_SV = swe.modulus((1,2,1))

#%% creaate pdf images of the different functions
#for var in [stress, mu_lt, mu_tl, mu_SH, mu_SV]:
swe.save_image(stress, '../figures/SI/stress.pdf')
swe.save_image(mu_tt, '../figures/SI/mu_tt.pdf')
swe.save_image(mu_lt, '../figures/SI/mu_lt.pdf')
swe.save_image(mu_tl, '../figures/SI/mu_tl.pdf')
swe.save_image(mu_SH, '../figures/SI/mu_SH.pdf')
swe.save_image(mu_SV, '../figures/SI/mu_SV.pdf')
```

5. Python Code Used to Generate Figures

The following Python files generate the data-containing figures in the main paper.

5.1. Figure 4

```
# fig04.py
# %%
import matplotlib.pyplot as plt
import numpy as np
import pandas as pd
import swe
from glob import glob


#% Figure 4
parms = swe.parms
plt.close('all')
fig, ax = plt.subplots(1, 2, figsize=(7,3), constrained_layout = True)
ax[0].set_xlabel(r'$\lambda_t$')
ax[1].set_xlabel(r'$\lambda_\ell$')
for k in [0,1]:
    ax[k].set_ylabel(r'$\sigma_N$ (kPa)')

axnum = {'f':1,  #longitudinal extension
         't':0}  #transverse extension

for geom in ['f', 't']:
```

```
    files = sorted(glob(f'../data/tensile/{geom}_*mm'))
    for file in files:
        samp = file[-5:-2]
        dL = float(samp[:3])
        ori = file[-10:-5]
        df = pd.read_csv(file, delimiter='\t', index_col=False, skiprows=23,
                         names=['t', 'l', 'F', 'strain', 'stress'],
                         encoding='windows-1252')
        df['lam'] = df['l']/df['l'][0]  # extension ratio
        ax[axnum[geom]].plot(df['lam'], df['stress']/1000, linewidth=1)

lamf = np.linspace(1, 1.25, 100)
lamt = np.linspace(1, 1.3, 100)

# dump plot for solid lines
ax[0].plot([], [], '-', color='gray', label='Exp. data in colors')
ax[1].plot([], [], '-', color='gray', label='Exp. data in colors')
# reset the property cycle
ax[0].set_prop_cycle(None)
ax[1].set_prop_cycle(None)

stress = swe.stress((2,2))[2, 2].subs(swe.delta, 1)
for parms['c_2'], parms['c_4'], ls in zip ([0, 0, -0.43], [0, 18.6, 18.6],
    [':', '-.', '--']):
    stress_func = swe.make_plotable(stress, [swe.laml], parms)

    ax[1].plot(lamf, 0.001*stress_func(lamf)/lamf, ls,
               label = f'$c_2$={parms["c_2"]}, $c_4$={parms["c_4"]}')
    ax[0].plot(lamt, 0.001*swe.transverse_extension(lamt, parms)/lamt, ls,
               label = f'$c_2$={parms["c_2"]}, $c_4$={parms["c_4"]}')

for k in [0,1]:
    ax[k].set_xlim(left = 1)
    ax[k].set_ylim(bottom=0)
    ax[k].legend(framealpha=0)

ax[0].set_title(r'a) transverse extension ($\perp$)')
ax[1].set_title(r'b) longitudinal extension ($\parallel$)')

fig.show()
fig.savefig('../figures/fig04.pdf')
# %%
```

5.2. Figure 5

```
# fog05.py
# %%
import matplotlib.pyplot as plt
```

```
import numpy as np
import pandas as pd
import swe

parms = swe.parms
#Figure 5,  plot the indentation curves

# ignore error from read_excel command
import warnings
warnings.filterwarnings('ignore', category=UserWarning, module='openpyxl')

plt.close('all')

# load data into a dictionary of dataframes, with keys corresponding to
    sheet
# names
dfs = pd.read_excel('../data/indentation.xlsx', sheet_name=None, index_col
    =0)

fea_data = pd.read_csv('../data/FEA_indentation_data.csv')
fea_s = pd.read_csv('../data/FEA_indentation_contact_moduli.csv')

# add normalized data to the dataframe
for k, df in dfs.items():
    # normalize displacement with the small dimension of the indenter
    df['nd'] = df.shifted_d_mm/parms['a']
    df['nP'] = 0.001*df.P_mN  / (2*parms['a']*parms['l'])

colors = ['tab:blue', 'tab:orange', 'tab:green', 'tab:red']
# specify the extension ratios
lamf = np.array([1, 1.026, 1.052, 1.078])  # extension ratio

shear_type = 'lt'
if shear_type == 'lt':
    col = 's_T'
elif shear_type == 'tl':
    col = 's_L'

fig, ax = plt.subplots(1, 2, figsize=(9,3), constrained_layout = True)

# plot indentation data
for k, df in dfs.items():
    idx_max = df[['nP']].idxmax()['nP']
    df_load = df[:idx_max]/1000
    i, ori = int(k[0]), k[1]
    t = df['t']

    if ori == 'x':  # perpendicular data
```

```
        label=f'{lamf[i]:.3f}' + r' $\perp$'
        ax[0].plot(df_load['nd'], df_load['nP'], color=colors[i],
                   label=label, linestyle = '-')
        s = fea_s.query('nd==0.02')[col].iloc[i]*1e-3 # contact modulus
    from FEA at d/a = 0.02
        ax[1].plot(df_load['nd'], df_load['nP']/s, color=colors[i],
                   label=label, linestyle = '-')
    else:  # parallel data
        label=f'{lamf[i]:.3f}' + r' $\parallel$'
        ax[0].plot(df_load['nd'], df_load['nP'], color=colors[i],
                   label=label, linestyle = '--')

# plot FEA data: shifted for clarity
for geom in ['L', 'T']:
    for i, disp_gel in enumerate(fea_data.disp_gel.unique()):
        df = fea_data.query('disp_gel==@disp_gel & geom==@geom')
        shift = 0.15 if geom == 'L' else -0.15  # shift data on x-axis for
    clarity
        ax[0].plot(df.nd+shift, df.stress*1e-3, ':', color=colors[i]) # in
    kPa

ax[0].legend(framealpha=0, title=r'$\lambda_{\ell}$', ncol=2, loc=4,
          columnspacing=.0, labelspacing=.0, handleheight=2.1,
          handlelength=1.4)

ax[1].legend(framealpha=0, title=r'$\lambda_{\ell}$', ncol=1,
          handleheight=2.1, labelspacing=.0,
          handlelength=1.4)


ax[0].set_xlabel(r'$\delta/a$')
ax[1].set_xlabel(r'$\delta/a$')

ax[0].set_ylabel(r'$\sigma_N$ (kPa)')
ax[1].set_ylabel(r'$\sigma_N/s_\perp$')

ax[0].annotate('',
               xy=(0.45, 85),
               xytext=(0.18, 85),
               textcoords='data',
               arrowprops=dict(arrowstyle="<-", shrinkB=0),
               ha='right', va='center')
ax[0].text(0.2, 90, r'$\lambda_{\ell}$', ha='left', va='center')

ax[0].annotate(r'$\perp$',
               xy=(0.4, 70),
               xytext=(0.64, 85),
               textcoords='data',
```

```
                arrowprops=dict(arrowstyle='->', connectionstyle="arc3,rad=-0.2", color='gray', shrinkA=0),
                ha='center', va='bottom')
ax[0].annotate(r'$\parallel$',
                xy=(1.0, 70),
                xytext=(0.84, 85),
                textcoords='data',
                arrowprops=dict(arrowstyle='->', connectionstyle="arc3,rad=-0.2", color='gray', shrinkA=0),
                ha='right', va='bottom')

ax[0].annotate(r'FEA, $\perp$',
                xy=(0.05, 70),
                xytext=(-0.20, 85),
                textcoords='data',
                arrowprops=dict(arrowstyle='->', connectionstyle="arc3,rad=0.2", color='gray', shrinkA=0),
                ha='left', va='bottom')
ax[0].annotate(r'FEA, $\parallel$',
                xy=(0.46, 14),
                xytext=(0.40, -6),
                textcoords='data',
                arrowprops=dict(arrowstyle='->', connectionstyle="arc3,rad=0.2", color='gray', shrinkA=0),
                ha='center', va='bottom')

# add lines  y = x + b
ax[1].plot([0.2, 0.4], [0.2-0.08, 0.4-0.08], 'k--')
# add the slope
ax[1].plot([0.25,0.35], [0.25-0.08, 0.25-0.08], 'k-')
ax[1].plot([0.35,0.35], [0.35-0.08, 0.25-0.08], 'k-')
ax[1].text(0.36, 0.3-0.08, r'1', ha='left', va='center', fontsize=12)

ax[0].set_xlim(-0.25, 1.6)
ax[1].set_xlim(-0.1, 0.5)

ax[0].set_title('(a)')
ax[1].set_title('(b)')

fig.show()
fig.savefig('../figures/fig05.pdf')
```

5.3. *Figure 6*

```
# fig06.py
import matplotlib.pyplot as plt
import swe
import pandas as pd

```

```
# Figure 6
plt.close('all')
file = '../data/FEA_gel_geom.xlsx'

df = pd.read_excel(file)

# set the range of x and y values to include and make the plot
xrange=[1,10]
yrange=[1,10]
fig, ax = swe.make_s_ratio_plot(df,  xrange, yrange)

# now draw line corresponding to values that match measured ratio
s_ratio = 3.65
s_ratio_err = 0.1
x_val = swe.parms['E_L']/(3*swe.parms['mu_T'])
x_err = 0.1
y_val = swe.fixed_s_ratio_func(df, s_ratio)(x_val)
y_err = swe.mu_par_error(df, x_val, x_err, s_ratio, s_ratio_err)

# plot lines corresopnding to different contact stifness ratios
rs = {0:s_ratio, # this is the actual value of r
     -1:(1-s_ratio_err)*s_ratio, # this is the lower limit of r, given the
    error
      1:(1+s_ratio_err)*s_ratio}  # this is the upper limit of r, given
   the error

fmt = {-1:'--r',
        0:'-k',
        1:'--b'}

for k in [-1, 0, 1]:
    swe.add_s_ratio_line(df, ax, rs[k], fmt[k])

# now plot the experimentl point, with the relevant error bars

ax.errorbar(x_val, y_val, xerr=x_err*x_val, yerr=y_err, fmt='ok',
            markersize=8, linewidth=2, capsize=10)

ax.legend(framealpha=0, labelspacing=0.1)
fig.show()
fig.savefig('../figures/fig06.pdf')
fig.savefig('../figures/fig06.jpg', dpi=1000)
```

5.4. Figure 7

```
# fig07.py
import matplotlib.pyplot as plt
import numpy as np
import swe
```

```
5
6  # plot normalized differential moduli (Figure 7)
7  plt.close('all')
8  parms = swe.parms
9
10 norm_mod_labels = {(2,2): r'$E_{ff}/E_L$',
11                   (0,1): r'$\mu_{tt}/\mu_T$',
12                   (0,2): r'$\mu_{tf}/\mu_L$',
13                   (2,1): r'$\mu_{ft}/\mu_L$'}
14
15 fig, ax = plt.subplots(1,2, figsize=(8,3), constrained_layout = True)
16 ax[0].set_xlabel(r'$\lambda_\ell$')
17 ax[1].set_xlabel(r'$\sigma_\ell$ (kPa)')
18
19 laml = np.linspace(1,1.1, 20)
20 stress=swe.stress((2,2))[2,2].subs(swe.delta, 1)
21 stress_func = swe.make_plotable(stress, [swe.laml], parms)
22 mod_func = {}
23 for idx in [(0,1), (0,2), (2,1), (2,2)]:
24     mod = swe.modulus(idx)
25     mod_func[idx] = swe.make_plotable(mod, [swe.laml], parms)
26     ax[0].plot(laml, mod_func[idx](laml)/mod_func[idx](1),
27                label = norm_mod_labels[idx])
28     ax[1].plot(stress_func(laml)/1000, mod_func[idx](laml)/mod_func[idx
       ](1),
29                label = norm_mod_labels[idx])
30
31 ax[1].plot(stress_func(laml)/1000, (stress_func(laml)+mod_func[(2,1)](laml
       ))/
32            mod_func[(2,1)](1), 'C1+',
33            label = r'$(\sigma_\ell+\mu_{\ell t})/\mu_T$')
34
35 for k in [0,1]:
36     ax[k].set_ylabel('Normalized modulus')
37     ax[k].legend(loc=2, framealpha=0)
38     ax[k].legend(loc=2, framealpha=0)
39 fig.show()
40 fig.savefig('../figures/fig07.pdf')
```

5.5. Figures 9 and 10

```
1  # fig09_10.py
2  import matplotlib.pyplot as plt
3  import numpy as np
4  import swe
5
6  # figure 9, figure 10
7  parms = swe.parms
8  '''
```

```
make the functions to look at the angular dependence of the
horizontal and vertical mode velocities.  Python is doing a lot of work
here and it will likely take a few minutes to generate the functions
The existence of any third number in the tuple argument of swe.modulus
means that we want to calculate the full theta dependence.
'''

print('calculateing theta-dependent shear moduli - be patient')
mu = {'SH': swe.modulus((0,2,1)),
      'SV': swe.modulus((1,2,1))}

#%%
# polar plots of the moduli, using functions generated above
plt.close('all')
# plot the incremental moduli/phase velocities
fig, ax = plt.subplots(1,2, figsize=(7, 3), constrained_layout = True,
                       subplot_kw={'projection': 'polar'})

# plot the group veolocities
fig2, ax2 = plt.subplots(1,2, figsize=(7, 3), constrained_layout = True,
                       subplot_kw={'projection': 'polar'})

theta = np.linspace(0, 2*np.pi, 360)

for laml in [1, 1.2]:
    parms['lambda_ell'] = laml
    for mode, axnum, label in zip(['SH','SV'], [0,1],
                                  [r'$\lambda_\ell = $' + f'{laml}', ''])
    :
        mod_func = swe.make_plotable(mu[mode], [swe.theta], parms)
        ax[axnum].plot(theta, mod_func(theta)/1000, label = label)
        vg, thetag = swe.group_velocity(mu[mode])
        vg_func = swe.make_plotable(vg, [swe.theta], parms)
        thetag_func = swe.make_plotable(thetag, [swe.theta], parms)
        ax2[axnum].plot(thetag_func(theta),
                        (1000)**(-0.5)*vg_func(theta), label = label)


for axval in [ax,ax2]: axval[0].legend(bbox_to_anchor=(1.5, 0.8), loc='
    center')
ax[0].set_title(r'(a) $\rho v_{SH}^2=\mu_{SH}$ (kPa)', pad=25)
ax[1].set_title(r'(b) $\rho v_{SV}^2=\mu_{SV}$ (kPa)', pad=25)

ax2[0].set_title(r'(a) $V_{SH} \rho^{1/2}\:$'+
                 r'$\left(m/s\cdot \left(g/cm^3\right)^{1/2}\right)$', pad
    =25)
ax2[1].set_title(r'(b) $V_{SV} \rho^{1/2}\:$'+
                 r'$\left(m/s\cdot \left(g/cm^3\right)^{1/2}\right)$', pad
```

```
   =25)
fig.show()
fig2.show()
fig.savefig('../figures/fig09.pdf')
fig2.savefig('../figures/fig10.pdf')
```

5.6. Functions for Elastic Analysis (swe.py)

```
# swe.py
from sympy import (symbols, Matrix, diff,
                   sqrt, eye, lambdify, simplify,
                   solve, exp, sin, cos, atan, latex, preview)
import numpy as np
from sympy.physics.quantum import TensorProduct
import scipy.optimize as optimize
from scipy.interpolate import LinearNDInterpolator, interp1d
import matplotlib.pyplot as plt

# display and preview not used here, but are handy to make available when
# calling the swe module
from IPython.display import display

# material parameters
# keep evertying in SI units to avoid confusion
parms = {
         r'mu_L': 183e3,   # 'paralllel' shear modulus
         r'mu_T': 49.7e3, # 'perpendicular' shear modulus
         r'E_L': 0.942e6, # 1e6 # 'parallel' extensional modulus
         'c_2': -0.43,  # isotropic strain hardening parameter
         'c_4': 18.6, # strain hardening parameter for fiber extension
         'a': 7e-5, # indenter half-width
         'h': 1.1e-3, # material thickness
         'l': 0.02, # indenter length
         'fr_s':0.1, # fractional error in contact stiffness ratio
         'fr_E':0.1} # fractional error in E_par/mu_T


# invariants
i1, i4, i5, = symbols(['I_1', 'I_4', 'I_5'])

# strains
laml, delta = symbols(['lambda_ell', 'delta'],  positive = True)

# symbolic forms of various incremental moduli
mutt, mutl, mult =symbols(['mu_tt', 'mu_t\ell', 'mu_\ellt'])

# symbolic forms for angle used to define group velocity
thetag = symbols('theta_g', real=True)

```

```
# parameters used in HGY formu_paration
c2, c4, mu_T, mu_par, E_par, beta = symbols(['c_2', 'c_4', r'mu_T',
            r'mu_L', r'E_L',  'beta'], positive = True)

# rotation angle arond 1 axis for incremental strain
theta = symbols('theta', real=True)

# define various ratios used in contact stiffness equations
a, h = symbols(['a', 'h'])

# hydrostatic pressure term
p = symbols('p')

# vector pointing along fiber axis
m = Matrix([[0],[0],[1]])

# strain energy funtion from  Hegde et al. Int. J. of Non-Linear Mech.
# 160, 104663 (2024) (http://dx.doi.org/10.1016/j.ijnonlinmec.2024.104663)
W =((mu_T/(2*c2))*(exp(c2*(i1-3))-1)+((E_par+mu_T-4*mu_par)/(2*c4))*
                (exp(c4*(sqrt(i4)-1)**2)-1) + ((mu_T-mu_par)/2)*(2*i4-i5
   -1))

# F for extensional prestrain
F0 = (Matrix([[1/sqrt(laml), 0, 0],
              [0, 1/sqrt(laml), 0],
              [0, 0, laml]]))

# rotation matrix for rotation of theta degrees around axis 0
R = Matrix([[1, 0, 0],
           [0, cos(theta), -sin(theta)],
           [0, sin(theta), cos(theta)]])

def F(idx):
    '''
    F is the deformation gradient tensor.  idx is a tuple describing the
    deformation: 1st number is displacement direction, 2nd is gradient
   direction.
    example values of idx are: (descriptions are with theta = 0)
    (1,1) or (1,1) transverse extension (laml = 1 only)
    (2,2) longitudinal extension
    (0,1) vertical shear propagating in transverse direction
    (0,2) vertical shear propgating in fiber direction
    (1,2) horizontal shear propagating in fiber direction
    '''

    F_inc = (Matrix([[1, 0, 0],
                     [0, 1, 0],
                     [0, 0, 1]]))
```

```
88
89      F_inc[idx[0],idx[1]] = delta # this is the increment to F
90
91      if idx==(0,0):  # transverse extensiont, laml = 1 by definition
92          F_inc[1,1] = sqrt(1/(beta*delta))
93          F_inc[2,2] = sqrt(beta/delta)
94
95      if idx==(1,1):  # transverse extensiont, laml = 1 by definition
96          F_inc[0,0] = sqrt(1/(beta*delta))
97          F_inc[2,2] = sqrt(beta/delta)
98
99      if idx==(2,2): # extension along fiber axes
100         F_inc[0,0] = sqrt(1/delta)
101         F_inc[1,1] = sqrt(1/delta)
102
103     # we put a third element in the idx tuple if we want to use the angle
104     if len(idx)>2:
105         F_inc = R*F_inc*R.transpose()
106
107     return F_inc*F0
108
109 def C(F):
110     # right Cauchy-Green Tensor
111     return F.transpose()*F
112
113 def C2(F):
114     return C(F)*C(F)
115
116 def B(F):
117     # left Cauch-Green Tensor
118     return F*F.transpose()
119
120 def I4(F):
121     # fourth strain invariant for fiber directed along x3
122     return m.dot(C(F)*m)
123
124 def I5(F):
125     # fifth strain invariant for fiber directed along x3
126     return  m.dot((C(F)*C(F))*m)
127
128 def I1(F):
129     # first strain invariant
130     return B(F).trace()
131
132 idx = np.arange(3) # used for nested loops
133
134 def stress(idx):
135     # Ogden Eq. 2.47
```

```
    tp4 = TensorProduct(F(idx)*m, F(idx)*m).reshape(3,3)
    tp5 = (TensorProduct(F(idx)*m, B(F(idx))*F(idx)*m).reshape(3,3)+
           TensorProduct(B(F(idx))*F(idx)*m, F(idx)*m).reshape(3,3))
    W1 = diff(W, i1)
    W4 = diff(W, i4)
    W5 = diff(W, i5)
    sigma = -p*eye(3) + 2*W1*B(F(idx))+2*W4*tp4+2*W5*tp5

    pval = solve(sigma[0,0], p)[0]
    sigma = sigma.subs(p, pval)
    sigma = sigma.subs(i1, I1(F(idx)))
    sigma = sigma.subs(i4, I4(F(idx)))
    sigma = sigma.subs(i5, I5(F(idx)))
    return simplify(sigma)

def modulus(idx):
    # calculate differential modulus from stress function
    if len(idx)>2:
        stress_tensor= R.transpose()*stress(idx)*R
    else:
        stress_tensor = stress(idx)
    stress_component = stress_tensor[idx[0], idx[1]]
    mod = diff(stress_component, delta)
    if idx[0]!=idx[1] : # don't need to do this for the extension cases
        mod = mod.subs(delta, 0)
    else:
        mod = mod.subs(delta, 1)
    mod = simplify(mod)
    return mod

def make_plotable(function, xvars, parms):
    '''
    function is a symbolic function of many variables
    this returns a lambdified version of the function as a function of
    the symbol variables in the  list xvars, substituting the other ones
    for the values in the parms dictionary
    '''
    variables = list(function.free_symbols)
    for xvar in xvars:
        try:
            variables.remove(xvar)
        except:
            print(f'problem removing {xvar}')
    for var in variables:
        try:
            function = function.subs(var, parms[var.name])
        except:
            print(f'problem substituting for {var}')
```

```
184
185     plot_function = lambdify(xvars, function, modules=["numpy"])
186     return plot_function
187
188 def guess_beta(lamtval, parms):
189     # low strain value for beta, used as initial guess calc_beta
190     return 1+(lamtval-1)*(1-4*parms[r'mu_T']/(parms[r'E_L']+
191                                                 parms[r'mu_T']))
192
193 def calc_beta(lamtval, parms):
194     '''
195     determine the value of beta that ensures all lateral normal stresses
    are
196     zero.  The stress function has the other transverse stress being zero,
197     so this function insures that the longitudinal stress is zero
198     '''
199     sigma = stress((1,1))[2,2]
200     sigma = sigma.subs(delta, lamtval)
201     ftosolve = make_plotable(sigma, [beta], parms)
202     guess = guess_beta(lamtval, parms)
203     soln = optimize.least_squares(ftosolve, guess, bounds=(0.5*guess, 2*
    guess))
204     return soln['x']
205
206 def transverse_extension_single(lamtval, parms, **kwargs):
207     # returns true stress for extension in transverse direction
208     # change beta_calc to True in call if you want to
209     # rigorously calculate beta for transverse extension
210     beta_calc = kwargs.get('beta_calc', False)
211     if beta_calc:
212         beta_val = calc_beta(lamtval, parms)[0]
213     else:
214         beta_val = guess_beta(lamtval, parms)
215     sigma = stress((1,1))[1,1]
216     sigma = sigma.subs(beta, beta_val).subs(laml, 1)
217     stress_func = make_plotable(sigma, [delta], parms)
218     return stress_func(lamtval)
219
220 def group_velocity(mu):
221     # input here is mu (either SH or SV)
222     v = sqrt(mu)
223     v2 = v*sin(theta)+diff(v, theta)*cos(theta)
224     v3 = v*cos(theta)-diff(v, theta)*sin(theta)
225     vg = sqrt(v2**2+v3**2) # group velocity
226     thetag = theta + atan(diff(v,theta)/v) # angle of propagation
227     return vg, thetag
228
229 transverse_extension = np.vectorize(transverse_extension_single)
```

```
calc_beta_vec = np.vectorize(calc_beta)
guess_beta_vec = np.vectorize(guess_beta)

# Now we have some functions related to the indentation experiments
# specify the varables
rs = symbols (r'r_s', real=True, positive=True) #s_perp/s_parallel
alpha, beta, A = symbols(['alpha', 'beta', 'A'])
rmu = symbols('r_mu', real=True, positive=True) #mu_L/mu_T
rE = symbols('r_E', real=True, positive = True)
frs, frE = symbols(['fr_s', 'fr_E'], real = True, positive =True)

def mu_par_error(df, x_val, x_err, s_ratio, s_ratio_err):
    # create a dictionary for the relvant values of the stiffness ratio
    s = {0:s_ratio, # this is the actual value of r
        -1:(1-s_ratio_err)*s_ratio, # this is the lower limit of r, given the error
         1:(1+s_ratio_err)*s_ratio}  # this is the upper limit of r, given the error

    # now we define a similar dictionary for the relevant values of the
    # normalized value of E_parallel
    x = {0:x_val, # this is the actual value of r
        -1:(1-x_err)*x_val, # this is the lower limit of r, given the error
         1:(1+x_err)*x_val}  # this is the upper limit of r, given the error

    yval = {}
    for s_idx in [-1, 0, 1]:
        yval[s_idx]={}
        for x_idx in [-1, 0, 1]:
            yval[s_idx][x_idx]=fixed_s_ratio_func(df,s[s_idx])(x[x_idx])

    # calculate error from uncertinty in s values
    yerr_s=[[yval[0][0]-yval[-1][0]], [yval[1][0]-yval[0][0]]]
    yerr_s = np.array(yerr_s)

    # now do the same thing for error from uncertainty in x (x is normallized E_par)
    yerr_x=[ [yval[0][0]-yval[0][-1]], [yval[0][1]-yval[0][0]]]
    yerr_x = np.array(yerr_x)

    return (yerr_x**2+yerr_s**2)**(0.5)

def ah_corr(ah):
    # ah here is the a/h ratio
    return -0.27 *np.log(ah/2)

```

```
def s_parallel(laml_vals, parms):
    # returns parallel contact modulus for different pre-strains
    ah = parms['a']/parms['h']
    mu_Tt = make_plotable(modulus((0,1)), [laml], parms)(laml_vals)
    s_parallel = mu_Tt/ah_corr(ah)
    return s_parallel

def s_perp(laml_vals, df, parms, shear_type):
    '''
    Calculate contact modulus for indenter aligned perpendicular to
    symmetry axis.

    Parameters
    ----------
    laml_vals : array of floats
        extension ratio in fiber direction.

    df : dataframe
        dataframe with FEA data used to calculate the contact stiffnesses.

    parms : dictionary
        parameter dictionary containingthe elastic constants.
    shear_type : string
        'tl' or 'lt' - generally assumed to be tl in the paper

    Returns
    -------
    s_perp : array of floats
        low-strain contact stiffness useing mu_tl or mu_lt as the shear
    modulus.
    '''

    Ell = make_plotable(modulus((2,2)), [laml], parms)(laml_vals)
    mu_T = make_plotable(modulus((0,1)), [laml], parms)(laml_vals)
    if shear_type=='tl':
        mu_par = make_plotable(modulus((0,2)), [laml], parms)(laml_vals)
    elif shear_type=='lt':
        mu_par = make_plotable(modulus((2,0)), [laml], parms)(laml_vals)
    else:
        print(f'shear_type of {shear_type} is not valid')
        return
    x = Ell/(3*mu_T)
    y = mu_par/mu_T

    rs=s_ratio_func(df)(x,y)

    s_perp = s_parallel(laml_vals, parms)*rs
    return s_perp
```

```
319
320 def print_latex(expression):
321     # print the latex code for the specified expression
322     latex_expression = latex(expression)
323     print(latex_expression)
324
325 def save_image(expression, filename):
326     with open(filename, 'wb') as outputfile:
327         preamble = "\\documentclass[10pt]{standalone}\n" \
328                    "\\usepackage{amsmath,amsfonts}\\begin{document}"
329         preview(expression, viewer='BytesIO', outputbuffer=outputfile,
330                 output = 'pdf', preamble=preamble)
331
332 def get_ah_values(df):
333     '''
334     Extract all a/h values from the FEA data into a list
335
336     Parameters
337     ----------
338     df : dataframe
339         Input dataframe
340
341
342     Returns
343     -------
344     List of all unique a/h values
345
346     '''
347
348
349     return   df['ah'].unique().tolist()
350
351 def choose_ah(df, ah):
352     '''
353     Refine fea dataframe to only include specified value within 10% of
    specified
354     value.
355
356     Parameters
357     ----------
358     df : dataframe
359         Iinput dataframe that may contain lots of a/h values
360
361     Returns
362     -------
363     Dataframe limited to specified a/h value.
364     '''
365     df['ah'] = df['a']/df['h']
```

```
    idx = df.index[abs(df.ah-ah)/ah<0.1].tolist()
    df_prop = df.loc[idx]
    return df_prop


def s_ratio_func(df):
    '''
    Fitting function for contact stiffness ratio, obtained from FEA data.

    Parameters
      ----------
      df : dataframe
          input dataframe with FEA data - should have single value of a/h

      Returns
      -------
      Function
          function of x, y, where x is E_parallel/3mu_T and y
          is mu_parallel/mu_T.
    '''

    def loginter2d(x, y, z):
        # function definition to create 2d interpolation in log domain for
    x,y
        logx = np.log10(x)
        logy = np.log10(y)
        linzfunc = LinearNDInterpolator(list(zip(logx, logy)), z)
        logzfunc = lambda a, b : linzfunc(np.log10(a), np.log10(b))
        return logzfunc

    # each value of a/h has a different function, which we put in a zfunc
   dict.

    x = df['Ef3Gt']  # this is our x axis variable
    y = df['GfGt']  # we cycle over all values of ths property
    z = df['rs']

    # Create an interpolating function using LinearNDzfunc
    return loginter2d(x, y, z)


def fixed_s_ratio_func(df, s_ratio):
    '''
    Fitting function for mu_parallel/mu_T vs E_parallel/3mu_T at fixed
    value of the contact stiffness ratio, r_perp/r_parallel.

    Parameters
    ----------
```

```
    df : dataframe
        Input dataframe with FEA data, should have single value for both a
    /h,
        and l/a.

    rs : Contact stiffness ratio (s_perp/s_parallel).

    Returns
    -------
    Function
        function of x where x is E_parallel/3mu_T and y
        is .
    '''

    xmin = df.Ef3Gt.min()
    xmax = df.Ef3Gt.max()
    ymin = df.GfGt.min()
    ymax = df.GfGt.max()
    x = np.geomspace(xmin, xmax, 100)
    y = np.array([], dtype=float)
    sfunc = s_ratio_func(df)
    for xval in x:
        if (np.isnan(sfunc(xval, ymax)) or
            s_ratio>sfunc(xval, ymax) or
            s_ratio<sfunc(xval, ymin)):
            y = np.append(y, np.nan)
        else:
            def ftosolve(yval):
                return sfunc(xval, yval)-s_ratio
            soln = optimize.least_squares(ftosolve, ymax,
                                          bounds=(ymin, ymax))
            y = np.append(y, soln['x'])
    log_interp = interp1d(np.log10(x), y)
    return lambda x : log_interp(np.log10(x))


def make_s_ratio_plot(df, xrange, yrange):
    '''
    Make a plot of the contact modulus ratio (s_perp/s_parallel)

    Parameters
    ----------
    df : datframe
        input dataframe with FEA dat (single a/h value for all data)
    xrange : list of two floats
        range of x data to plot.
    yrange : TYPE
        range of y data to plot.
```

```
459
460     Returns
461     -------
462     Fig and ax handles for contour plot of stiffness ratio as a function
        of
463     E_parallel/3mu_T and mu_parallel/mu_T.
464     '''
465
466     zfunc = s_ratio_func(df)
467
468     fig, ax = plt.subplots(1,1, figsize = (4, 3), constrained_layout =
        True)
469
470     x_new = np.linspace(xrange[0], xrange[1], 300)
471     y_new = np.linspace(yrange[0], yrange[1], 300)
472
473     X_new, Y_new = np.meshgrid(x_new, y_new)
474     Z_new = s_ratio_func(df)(X_new,Y_new)
475
476     # Plot the interpolated function
477     image = ax.pcolormesh(X_new, Y_new, Z_new, cmap='viridis',
478                     vmin=zfunc(xrange[0], yrange[0]),
479                     vmax=zfunc(xrange[1], yrange[1]))
480     fig.colorbar(image, label = r'$s_\perp/s_\parallel$')
481
482
483     # Redefine format_coord
484     def format_coord(x_val, y_val):
485         z_val = zfunc(x_val, y_val)
486         return f'x={x_val:.2f}, y={y_val:.2f}, z={z_val:.2f}'
487
488     ax.format_coord = format_coord
489
490     ax.set_xlabel(r'$E_L/(3\mu_T)$')
491     ax.set_ylabel('$\mu_L/\mu_T$')
492     ax.set_xlim(xrange)
493     ax.set_ylim(yrange)
494
495     fig.show()
496
497
498     return fig, ax
499
500 def add_s_ratio_line(df, ax, s_ratio, fmt, **kwargs):
501     '''
502     Add line to the plot at specified value of s_ratio
503
504     Parameters
```

```
    ----------
    df : dataframe
        input dataframe with FEA data
    ax : axis handle
        axis to plot on.
    s_ratio : float
        falue of contact stiffness ratio corresponding to the line
    fmt : format strong for plotting the line

    Returns
    -------
    No return - just updates the axis by adding the line

    '''
    label = kwargs.get('label', fr'$s_\perp/s_\parallel$={s_ratio:.2f}')
    [xmin, xmax] = ax.get_xlim()
    [ymin, ymax] = ax.get_ylim()
    xr = np.linspace(xmin, xmax, 100)
    yr = fixed_s_ratio_func(df, s_ratio)(xr)
    ax.plot(xr, yr, fmt, label = label)
    ax.set_ylim([ymin, ymax])
    return
```

References

[1] Q. Wang, W. B. Griffith, M. Einsla, S. Zhang, M. L. Pacholski, K. R. Shull, Bulk and Interfacial Contributions to the Adhesion of Acrylic Emulsion-Based Pressure-Sensitive Adhesives, Macromolecules 53 (16) (2020) 6975–6983. doi:10.1021/acs.macromol.0c01354.

[2] Transversely Isotropic Material - OSUPDOCS.
URL https://osupdocs.forestry.oregonstate.edu/index.php/Transversely_Isotropic_Material

[3] R. Namani, Y. Feng, R. J. Okamoto, N. Jesuraj, S. E. Sakiyama-Elbert, G. M. Genin, P. V. Bayly, Elastic Characterization of Transversely Isotropic Soft Materials by Dynamic Shear and Asymmetric Indentation, Journal of Biomechanical Engineering 134 (6) (Jun. 2012). doi:10.1115/1.4006848.

[4] Y. Feng, C.-H. Lee, L. Sun, S. Ji, X. Zhao, Characterizing white matter tissue in large strain via asymmetric indentation and inverse finite element modeling, Journal of the Mechanical Behavior of Biomedical Materials 65 (2017) 490–501. doi:10.1016/j.jmbbm.2016.09.020.
